# Dual-radical-based molecular anisotropy and synergy effect of semi-conductivity and valence tautomerization in a photoswitchable coordination polymer

**DOI:** 10.1093/nsr/nwad047

**Published:** 2023-02-23

**Authors:** Jing-Wei Dai, Yu-Qin Li, Zhao-Yang Li, Hai-Tao Zhang, Carmen Herrmann, Shohei Kumagai, Marko Damjanović, Markus Enders, Hiroyuki Nojiri, Masakazu Morimoto, Norihisa Hoshino, Tomoyuki Akutagawa, Masahiro Yamashita

**Affiliations:** State Key Laboratory of Medicinal Chemical Biology, Nankai University, Tianjin300071, China; School of Materials Science and Engineering, Nankai University, Tianjin300350, China; School of Materials Science and Engineering, Nankai University, Tianjin300350, China; Institute of Inorganic and Applied Chemistry, University of Hamburg, Hamburg22761, Germany; Institute of Inorganic and Applied Chemistry, University of Hamburg, Hamburg22761, Germany; Department of Chemistry, Graduate School of Science, Tohoku University, Sendai980-8578, Japan; Institute of Inorganic Chemistry, University of Heidelberg, HeidelbergD-69120, Germany; Institute of Inorganic Chemistry, University of Heidelberg, HeidelbergD-69120, Germany; Institute for Materials Research, Tohoku University, Sendai980-8577, Japan; Department of Chemistry, Rikkyo University, Tokyo171-8501, Japan; Institute of Multidisciplinary Research for Advanced Materials (IMRAM), Tohoku University, Sendai980-8577, Japan; Institute of Multidisciplinary Research for Advanced Materials (IMRAM), Tohoku University, Sendai980-8577, Japan; Department of Chemistry, Graduate School of Science, Tohoku University, Sendai980-8578, Japan

**Keywords:** valence tautomerization, charge transfer, photo switching, diradical, coordination polymer

## Abstract

Organic radicals are widely used as linkers or ligands to synthesize molecular magnetic materials. However, studies regarding the molecular anisotropies of radical-based magnetic materials and their multifunctionalities are rare. Herein, a photoisomerizable diarylethene ligand was used to form {[Co^III^(3,5-DTSQ^·–^)(3,5-DTCat^2–^)]_2_(6F-DAE-py_2_)}·3CH_3_CN·H_2_O (**o-1**·3CH_3_CN·H_2_O, 6F-DAE-py_2_ = 1,2-bis(2-methyl-5-(4-pyridyl)-3-thienyl)perfluorocyclopentene), a valence-tautomeric (VT) coordination polymer. We directly observed dual radicals for a single crystal using high-field/-frequency (∼13.3 T and ∼360 GHz) electron paramagnetic resonance (EPR) spectroscopy along the *c*-axis, which was further confirmed by angle-dependent Q-band EPR spectroscopy. Moreover, a conductive anomaly close to the VT transition temperature was observed only when probes were attached at the *ab* plane of the single crystal, indicative of synergy between valence tautomerism and conductivity. Structural anisotropy studies and density functional theory (DFT) calculations revealed that this synergy is due to electron transfer associated with valence tautomerism. This study presents the first example of dual-radical-based molecular anisotropy and charge-transfer-induced conductive anisotropy in a photoswitchable coordination polymer.

## Teaser

A photoswitchable and valence tautomeric coordination polymer was synthesized and displayed significant molecular anisotropy that helped directly observing dual organic radicals. Synergy effect between magnetic bistability and conductivity was realized.

## INTRODUCTION

Reducing the scale of manufacturing processes using effective, small-sized alternative materials is a recent focus of scientific research, with the miniaturization of pure Si nanostructures according to Moore's law being almost complete [1]. Materials that exhibit different states in response to external stimuli on a single-molecule level are currently being scrutinized, as they represent the ultimate form of miniaturization [2,3]. Therefore, compounds that combine orbitally degenerate metals with organic radical ligands, including spin-crossover (SCO) [4,5] and valence-tautomeric (VT) compounds [6,7], are being increasingly explored as novel bistable magnetic materials. Since the first report on valence tautomerism in the 1980s [8], several octahedral Co complexes with redox-active 3,5-di-*tert*-butyldioxolene and N-donor ancillary ligands have been reported to undergo VT transitions [9–13]. In addition, molecular materials should ideally display abrupt transitions and magnetic susceptibility hystereses that result in bistability [14]. These characteristics may be accessed in cooperative systems, particularly 1D coordination polymers (CPs) [9]. Daisuke *et al.* successfully synthesized thin films of SCO CPs that exhibit large thermal hysteresis loops [15], while Guo *et al.* reported the auxiliary alkyl-chain-modulated SCO behavior of [Fe(H_2_Bpz_2_)_2_(C*_n_*-bipy)] (pz = pyrazolyl, C*_n_*-bipy = bipyridine alkyl chain diester, *n* = 3, 4 and 5) complexes [16]. Although considerable progress has been achieved with SCO CPs, VT CPs have received limited research attention [17].

Redox-active ligands that can switch between different oxidation states are critical components of VT compounds [18]. In addition to magnetic measurements [19,20] and electronic spectroscopy [21,22], electron paramagnetic resonance (EPR) spectroscopy [23,24] can be utilized to study valence tautomerism, as most redox-active ligands employed in VT compounds contain radicals. Generally, one clear signal is revealed by EPR spectroscopy owing to the existence of a single electron on the semiquinone radical SQ^·–^ in low-spin (LS) Co^III^(3,5-DTCat^2–^)(3,5-DTSQ^·–^) (3,5-DTCat^2–^ = 3,5-di-*tert*-butylcatecholate, 3,5-DTSQ^·–^ = 3,5-di-*tert*-butylsemiquinone) species. Investigating intrinsic radical properties by combining single-crystal X-ray diffraction (SC-XRD) and EPR methods can provide a comprehensive understanding of how molecular and electronic structures are correlated [25,26]. However, very few studies have focused on the directionalities of radicals and on anisotropies in molecular magnetic materials under ambient conditions [27–29], let alone in VT complexes. Exploring these aspects may also aid in clarifying the internal mechanisms of other physical properties.

In several cases, bifunctional VT compounds have been obtained by introducing ligands that exhibit fluorescence [30,31], conductivity [32,33] and photoisomerization [34]. Furthermore, electronic conductivity, a basic property of matter [35], is extensively used by various multifunctional materials [36–38]. Lukyanov *et al.* investigated the effect of metal–ligand electron transfer on the conductivities of two semiquinonate complexes [39]; however, conductivity/magnetism synergy was not analysed. O’Sullivan *et al.* attempted to synthesize a VT conductive metal polymer [40], while Kanegawa *et al.* synthesized a novel VT compound by covalently linking a conductive tetrathiafulvalene-functionalized (TTF-functionalized) phenanthroline ligand, but no relevant conductivity data were provided [33]. Compared with most SCO conductors, the electron-transfer process in the VT complex is intramolecular, indicating a potentially larger transfer rate. In addition, electron transfer occurs simultaneously with the valence tautomerism of the metal center in the complexes. This contributes to the study of the synergistic effect between magnetism and conductivity. Currently, studies on VT conductors are rare in relation to those on SCO conductors, and more conductive VT systems need to be developed. In particular, elucidating synergistic mechanisms within these bifunctional materials is of considerable significance owing to a lack of similar previous studies.

In this study, we synthesized two VT CPs, namely {[Co^III^(3,5-DTSQ^·–^)(3,5-DTCat^2–^)]_2_(6F-DAE-py_2_)}·3CH_3_CN·H_2_O (**o-1**·3CH_3_CN·H_2_O, 6F-DAE-py_2_ = 1,2-bis(2-methyl-5-(4-pyridyl)-3-thienyl)perfluorocyclopentene) and [Co^III^(3,5-DTSQ^·–^)(3,5-DTCat^2–^)(6F-DAE-py_2_)]·H_2_O (**c-1**·H_2_O). The directionalities of the crystallographically distinct radicals were confirmed by using angle-dependent EPR spectroscopy. Usually, directionality indicates a phenomenon in which specific properties of the complex differ when the observation is executed from different directions. Here, the specific property refers to the dual radical phenomenon observed in high-field and high-frequency single-crystal EPR spectroscopy. Furthermore, density functional theory (DFT) calculations were employed to investigate the mechanism of the conductivity/magnetism synergy observed in **o-1**·3CH_3_CN·H_2_O, which was expected to be derived from electron transfer associated with the VT transition. SC-XRD, magnetic studies, electronic spectroscopy and nuclear magnetic resonance (NMR) spectroscopy were also performed to analyse **o-1**·3CH_3_CN·H_2_O in detail.

## RESULTS AND DISCUSSION

### Strategy and chain structures

A pyridyl derivative of a diarylethene (DAE) ligand was used as the linker along with the Co-dioxolene unit, which is a classical VT building block, to form a VT CP with a chain structure. Light may promote the rearrangement of the crystal structure and further influence magnetic properties through modification of the ligand field (isomers differ substantially in shape and conjugation), leading to photocontrol and deep insight into organic radicals on a 1D chain. Here, we synthesized the novel VT **o-1**·3CH_3_CN·H_2_O CP using an open-form derivative of the DAE ligand. The NMR spectrum and SC-XRD structure of the open-form ligand are shown in Supplementary Fig. S1a and S1b. The 6F-DAE-py_2_ ligand isomerizes when irradiated with light and has been widely studied for the construction of photochromic or photoswitchable compounds. David *et al.* presented two erbium(III)-based coordination systems that showed slow dynamics of magnetization using 6F-DAE-py_2_ as bridging ligands [41]. A 1D coordination solid was also synthesized by reaction of the DAE photochromic unit with the dysprosium-based single-molecule magnet [42]. In other cases, the 6F-DAE-py_2_ ligand was used as the photoresponsive component in photoswitchable materials [43,44]. However, less attention has been given to the bending structure in space that may lead to specific properties, as depicted in this work. Here, the closed-form polymer (**c-1**·H_2_O) was formed using the closed-form ligand **c-L**, which was obtained from a 365-nm UV-light-irradiated solution of **o-L**.

**Figure 1.**
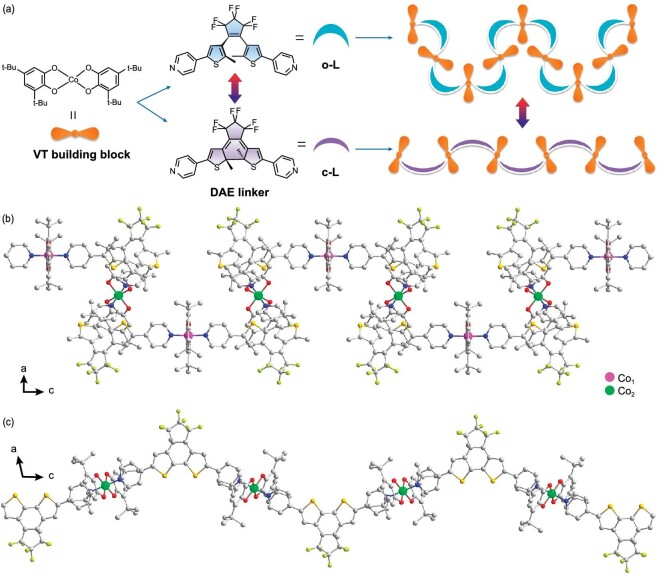
Schematic diagram and chain structures of **o-1**·3CH_3_CN·H_2_O and **c-1**·H_2_O. (a) Valence-tautomeric (VT) building block and diarylethene (DAE) linker that form zigzag **o-1**·3CH_3_CN·H_2_O and quasi-linear **c-1**·H_2_O; (b) zigzag chain in **o-1**·3CH_3_CN·H_2_O viewed along the *b*-axis; (c) quasi-linear chain in **c-1**·H_2_O viewed along the *b*-axis. Hydrogen atoms and solvent molecules are omitted for clarity. Color code: Co_1_, pink; Co_2_, green; O, red; N, blue; C, gray; S, orange; F, yellow−green.

Notably, **o-1**·3CH_3_CN·H_2_O and **c-1**·H_2_O exhibit the same coordination mode, in which each Co(III) center is coordinated to two dioxolene ligands (one in the catecholate state and the other in the semiquinone state) through four oxygen atoms, with two ancillary ligands located in the vertical direction with *trans*-disposed N atoms of pyridyl groups. The central Co ions are therefore octahedrally configured. Discrete Co-dioxolene units are connected by linear 6F-DAE-py_2_ ligands to form 1D chains along the *c*-axis. However, the 1D chains in **o-1**·3CH_3_CN·H_2_O and **c-1**·H_2_O differ due to the open and closed forms of the 6F-DAE-py_2_ ligand (Fig. 1a). Two terminal pyridines approach each other in **o-L**, rendering the open-form ligand a simplified ‘arc’; consequently, **o-1**·3CH_3_CN·H_2_O exhibits a zigzag 1D structure. The arc radian is smaller in **c-L** than in **o-L**; hence, **c-1**·H_2_O displays quasi-linear instead of zigzag characteristics. The asymmetric units of **o-1**·3CH_3_CN·H_2_O and **c-1**·H_2_O are shown in Supplementary Fig. S2, with crystallographic data shown in Supplementary Tables S1–S3. Powder XRD revealed pure phases (Supplementary Fig. S3).

**Figure 2.**
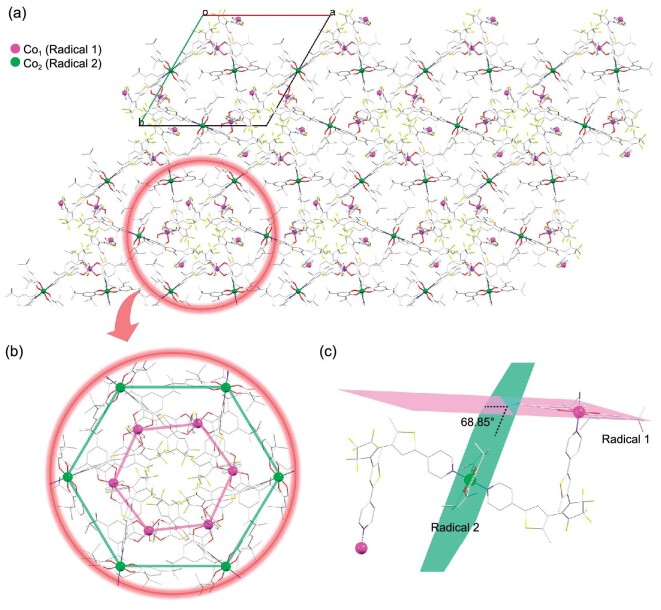
Packing diagrams of **o-1**·3CH_3_CN·H_2_O showing crystallographically distinct central Co ions. (a) Packing diagrams of **o-1**·3CH_3_CN·H_2_O viewed along the *c*-axis, showing two crystallographically distinct Co centers in pink and green; (b) hexagonal arrangements of the two types of Co centers in the *ab* plane; (c) detailed configurations of the two radicals coordinated to Co_1_ and Co_2_. Hydrogen atoms and solvent molecules are omitted for clarity. Color code: Co_1_, pink; Co_2_, green; O, red; N, blue; C, gray; S, orange; F, yellow−green.

Packing diagrams of **o-1**·3CH_3_CN·H_2_O and **c-1**·H_2_O are shown in Fig. 1b and c, with both exhibiting 1D chains. However, these chains differ due to the different configurations of **o-L** and **c-L. o-1**·3CH_3_CN·H_2_O crystallizes in the *P*6_3_/*m* space group, with the Co ions in distorted octahedral environments. Two types of crystallographically distinct central Co ions, referred to as Co_1_ and Co_2_, are observed in these chains due to their zigzag configurations. In **o-1**·3CH_3_CN·H_2_O, we may observe that the two types of central Co ions spread hexagonally over the *ab* plane when viewed along the *c*-axis (Fig. 2a and b, with Co_1_ and Co_2_ shown in pink and green, respectively); this corresponds to the hexagonal crystal system of the open-form complex. **o-1**·3CH_3_CN·H_2_O was subjected to SC-XRD at 100 K. The C−O bond lengths enable the Cat^2–^ (single bond: 1.344/1.355 Å, with the expected value in the range of 1.33−1.39 Å) and SQ^·–^ (double bond: 1.317/1.362 Å) states of the two dioxolene ligands coordinated to Co_1_ to be identified, whereas Co_2_ is located at an imposed inversion center, rendering Cat^2–^ and SQ^·–^ indistinguishable (Supplementary Fig. S1c and S1d). In this case, the electronic states of the dioxolene ligands of Co_2_ are averaged and random rather than directional electron transfer is observed between the ligand and metal center. Moreover, the steric configurations of the dioxolene ligands on Co_1_ and Co_2_ differ. Figure 2c reveals that the semiquinone ligand coordinated to Co_1_ (referred to as Radical 1) and the dioxolene ligand coordinated to Co_2_ (both referred to as Radical 2) are not coplanar, with a dihedral angle of 68.85° between them. The crystal structure modes used in the DFT calculations are described in Supplementary Figs S4−S6. In contrast, **c-1**·H_2_O crystallizes in the monoclinic *P*2_1_/*n* space group, with only one type of Co central ion in the chain connected by the less distorted **c-L**. Packing diagrams of **c-1**·H_2_O are shown in Supplementary Fig. S7. Additionally, SC-XRD helps to elucidate the oxidation states of the ligands and Co centers in **c-1**·H_2_O.

### Electronic and NMR spectroscopy

**Figure 3.**
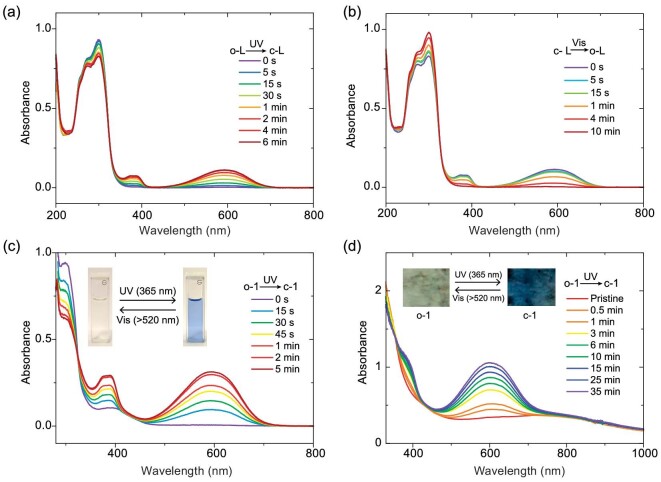
UV–visible absorption spectra of the ligand and complexes. Absorption spectra of the 6F-DAE-py_2_ ligand upon irradiation with (a) 365-nm UV light (handy thin-layer chromatography (TLC) lamp, power = 4 W) and (b) an LED flashlight for the 6F-DAE-py_2_ ligand; absorption spectra of **o-1**·3CH_3_CN·H_2_O upon irradiation with 365-nm UV light in (c) DMF solution and (d) in a solid-state KBr pellet. Insets: photographic images showing the photoisomerization between **o-1**·3CH_3_CN·H_2_O and **c-1**·H_2_O in the solution/solid state under UV or visible light.

Photoconversion between the open- and closed-form species (ligands and complexes) due to the effect of DAE ligand photoisomerization was explored. Reversible conversion of **o-L** and **c-L** under UV or visible light was confirmed by using UV/vis absorption spectroscopy, with second-timescale responses observed (Fig. 3a and b). Electronic absorption spectroscopy was then used to qualitatively study the photoconversion of **o-1**·3CH_3_CN·H_2_O and **c-1**·H_2_O when irradiated with light. Photocyclization of **o-1**·3CH_3_CN·H_2_O in dimethylformamide (DMF) is initiated by 365-nm UV light in seconds, with the solution observed to change from colorless to light blue. The reverse reaction occurs when irradiated with visible light (>520 nm, Fig. 3c). Photoisomerization was also studied in the solid state using a KBr pellet (Fig. 3d). Photocyclization occurred in minutes when irradiated with 365-nm UV light and was complete in one hour; the reverse process (from **c-1**·H_2_O to **o-1**·3CH_3_CN·H_2_O) was also observed under visible light. Despite the longer response times compared with those observed for pure organic DAE crystals [45] or single-molecule junctions [46], **o-1**·3CH_3_CN·H_2_O and **c-1**·H_2_O effectively photoswitch in solution or in the bulk state in a reversible manner.

NMR spectroscopy was used to further qualitatively and semiquantitatively study photocyclization in solution (Supplementary Figs S8−S13). ^1^H NMR spectroscopy was used to study the stability of **o-1**·3CH_3_CN·H_2_O upon heating. ^19^F NMR spectroscopy was used to track the photocyclization progress and calculate yields based on ^19^F signal intensities. To support the experimental results, we performed DFT calculations based on monomeric and dimeric models (Supplementary Figs S14−S18 and Supplementary Tables S4−S8). The expected sextet electronic states of the high-spin (HS) Co^II^(3,5-DTSQ^·–^)_FM_(3,5-DTSQ^·–^)_FM_ building blocks in the 1D **o-1**·3CH_3_CN·H_2_O complex were observed. The dominant species was found to be six-coordinate, with an average of approximately 190 Co metal centers in solution, based on the spin densities determined for these optimized structures. In addition, a comparison between the obtained spin densities of the five- and six-coordinate structures enabled the unambiguous identification of the main species represented in the ^1^H NMR spectrum of **o-1**·3CH_3_CN·H_2_O in DMF-d_7_.

### EPR spectroscopy

**Figure 4.**
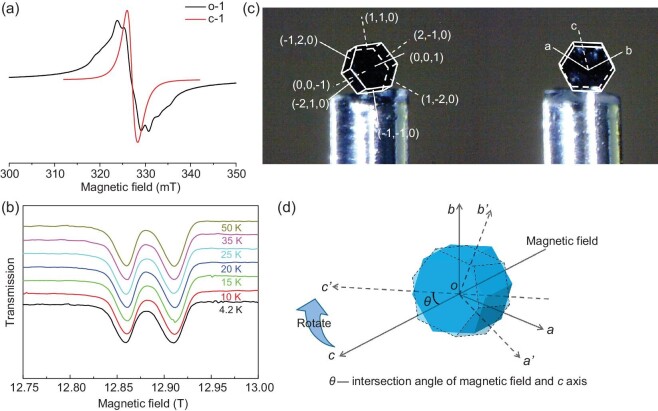
Electron paramagnetic resonance study of **o-1**·3CH_3_CN·H_2_O and **c-1**·H_2_O. (a) X-band electron paramagnetic resonance (EPR) spectra of **o-1**·3CH_3_CN·H_2_O and **c-1**·H_2_O powders acquired at 100 K; (b) high-field (∼13.3 T) and -frequency (∼360 GHz) single-crystal EPR spectra acquired at various temperatures with the *c*-axis along the static field; (c) face index of a single crystal of **o-1**·3CH_3_CN·H_2_O; (d) schematic diagram of single-crystal rotation during angle-dependent EPR spectroscopy.

Single crystals of **o-1**·3CH_3_CN·H_2_O and **c-1**·H_2_O exhibit significant structural anisotropies involving SQ^·–^ radicals at the Co metal centers. EPR spectroscopy can be used to confirm the presence of SQ^·–^ radicals in **o-1**·3CH_3_CN·H_2_O and **c-1**·H_2_O. The X-band (9.1 GHz) EPR spectra of powder samples of the open- and closed-form complexes at 100 K (Fig. 4a) are consistent with the presence of organic radicals (*g* ≈ 2.0023); broad lines that lack hyperfine features were observed, suggesting that the SQ^·–^ radical is immobilized due to coordination with the LS closed-shell Co(III) center (*S* = 0). Interactions between the spin magnetic moments of unpaired electrons and metal nuclei were absent in these systems and **o-1**·3CH_3_CN·H_2_O and **c-1**·H_2_O exhibited different degrees of line broadening. Subsequently, high-field (∼13.3 T) and -frequency (∼360 GHz) single-crystal EPR spectroscopy was used to significantly enhance the resolution of the Zeeman splitting in **o-1**·3CH_3_CN·H_2_O, which led to the observation of a remarkable phenomenon. In particular, we directly visualized dual SQ^·–^ radical signals that correspond to two crystallographically independent Co building blocks associated with the zigzag chain structure when the *c*-axis was aligned with the static field (Fig. 4b). Strong signals centered at 12.86 and 12.91 T with *g* = 2.000(1) and 1.992(3), respectively, were observed at 50 K. These dual radicals remained during cooling from 50 to 4.2 K. Moreover, only a split peak and no dual radical signals were observed when the static field was applied in other directions (Supplementary Fig. S19). Such directional observation of dual radicals is rare in VT systems [47,48]. Two crystallographically independent radicals are detected using high-field and high-frequency EPR spectroscopy along the *c*-axis because this axis corresponds to the extension direction of the 1D chains in **o-1**·3CH_3_CN·H_2_O. In contrast, no dual SQ^·–^ radical signals were observed for **c-1**·H_2_O due to its quasi-linear 1D structure.

To conveniently investigate the two directional radicals, we determined the face index of a single crystal of **o-1**·3CH_3_CN·H_2_O, as shown in Fig. 4c, with other face index diagrams shown in Supplementary Fig. S20. The crystal is regular hexagonal prismatic in shape, with the two subfaces having Miller indices of (001) and (00–1). The Miller indices of the six side faces are (110), (2–10), (1–20), (–1–10), (–210) and (–120). The single-crystal structure of **o-1**·3CH_3_CN·H_2_O suggests that the *c*-axis is perpendicular to the two subfaces and parallel to the hexagonal prism, i.e. the 1D chains in **o-1**·3CH_3_CN·H_2_O are arranged in the direction of the central rod of the hexagonal prism. The *c*-axis and *ab* plane form an angle of 90° as **o-1**·3CH_3_CN·H_2_O crystallizes in the hexagonal crystal system. Hence, the *ab* plane is parallel to the subface of the hexagonal prism.

**Figure 5.**
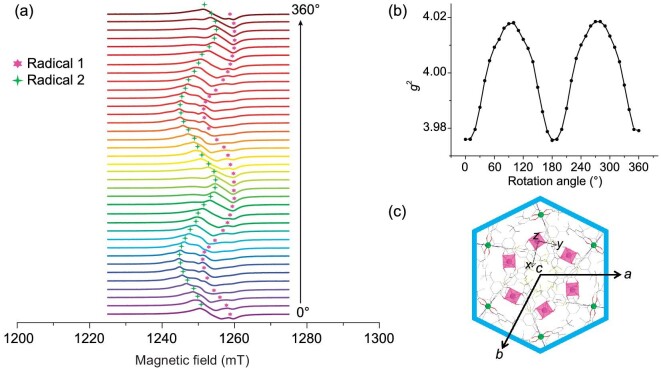
Angle-dependent electron paramagnetic resonance study of **o-1**·3CH_3_CN·H_2_O. (a) Angular dependences of the Q-band electron paramagnetic resonance spectra acquired at 300 K along the *c*-axis of a single crystal of **o-1**·3CH_3_CN·H_2_O (defined as 0°), revealing the well-resolved structural anisotropy of the **o-1**·3CH_3_CN·H_2_O single crystal; (b) angular dependence of the *g* factor of **o-1**·3CH_3_CN·H_2_O at 300 K with a rotating magnetic field; (c) locations of Co_1_ and Co_2_ in the *ab* plane with respect to the *c*-axis.

We used a single crystal of **o-1**·3CH_3_CN·H_2_O to collect a full rotational pattern of EPR spectra when the magnetic field was perpendicular to the *ab* plane to further study the two independent radical signals, which directly reflect the orientations of the radicals in the crystal frame. As shown in Fig. 4d, a static field is exerted along the *c*-axis, i.e. the magnetic field coincides perfectly with the *c*-axis. The intersection angle of the field and *c*-axis is defined as *θ* and was initially 0°. The single crystal was then rotated to move the *c*-axis close to the initial position of the *ab* plane; hence, *θ* increased gradually. EPR spectra were acquired as the angle was varied from 0° to 360°. The X-band EPR spectra of **o-1**·3CH_3_CN·H_2_O did not show a clear difference between the signals of the two radicals (Supplementary Fig. S21). However, the resolution of the Q-band (34.0 GHz) single-crystal EPR spectrum of **o-1**·3CH_3_CN·H_2_O acquired at 300 K is improved by approximately one order of magnitude, thereby providing higher sensitivity for distinguishing the two independent radicals as the angle was varied from 0° to 360° (Fig. 5a). Meanwhile, the angle-dependent behavior of the *g*^2^ value in the Q-band is consistent with these results (Fig. 5b). The *g*^2^ value exhibits a characteristic periodic variation from a minimum of 3.976(0) to a maximum of 4.017(8) as *θ* increases from 0° to 90° and then declines to 3.975(6) at *θ* = 180°. The angle-dependent EPR data for **o-1**·3CH_3_CN·H_2_O are likely to be associated with its crystal structure and the two independent radicals. The *c*-axis corresponds to the extension direction of the 1D chains in **o-1**·3CH_3_CN·H_2_O. The *g*_z_ of Co_1_ in the octahedral configuration can be identified in angle-dependent EPR spectroscopy incorporating the face index. This is due to the coincidence of *g*_z_ of Co_1_ and the *c*-axis. Notably, the *g*_1_ value corresponds exactly to the *g*_z_ value of Radical 1 at *θ* = 0° (the field is directed through two conical points of the Co_1_ octahedral sphere). The *g* values of 1.994(0) and 1.993(9) at *θ* = 0° and 180°, respectively, indicate that the *g* value of 1.992(3) obtained in the high-field and high-frequency spectra is attributed to Radical 1, *g*_1_. In this case, the *g*_2_ value of Radical 2 is 2.000(1), which is obtained in the high-field and high-frequency spectra. After the *c*-axis rotates into the initial *ab* plane (*θ* = 90°), where *g*^2^ realizes its maximum value, the measured *g* value of the Co_1_ center is *g*_xy_ (Fig. 5c). The field direction coincides with the *c*-axis again as the single crystal is continuously rotated. One periodic variation is completed as *θ* is increased from 0° to 180°.

### Magnetic studies

**Figure 6.**
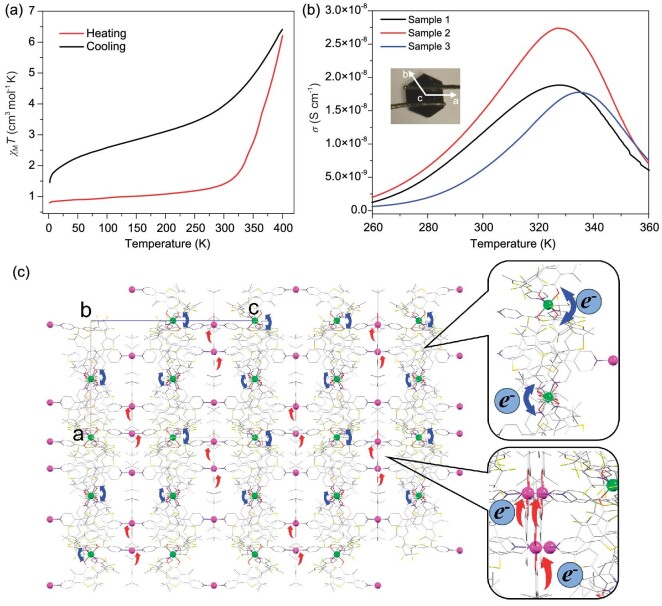
Studies of magnetic and conductive properties for **o-1**·3CH_3_CN·H_2_O. (a) Magnetic susceptibility of **o-1**·3CH_3_CN·H_2_O. The red curve corresponds to the heated fresh sample with crystal solvent and the black curve corresponds to cooling after solvent loss. Further cyclic measurements showed no hysteresis of dissolved **o-1**·3CH_3_CN·H_2_O. (b) Electrical conductivity of **o-1**·3CH_3_CN·H_2_O, with three single crystals used to confirm repeatability and exclude measurement errors. Inset: schematic diagram of the wiring method. (c) Side view of the short-range conductive pathways in the *ab* plane. Upper enlargement: random electron transfer at Co_2_ centers due to the averaged states of the dioxolene ligands. Lower magnification: directional ligand-to-metal electron transfer at Co_1_ centers. Hydrogen atoms and solvent molecules are omitted for clarity. Color code: Co_1_, pink; Co_2_, green; O, red; N, blue; C, gray; S, orange; F, yellow−green.

To evaluate how structural switching affects its physical properties, the magnetic susceptibility of **o-1**·3CH_3_CN·H_2_O was measured in the range of 5–400 K, the results of which are shown in Fig. 6a. *χ_m_T* (where *χ_m_* is the molar magnetic susceptibility and *T* is the temperature) was observed to be almost constant (approximately 0.9 cm^3^ mol^−1^ K) regardless of the temperature below 300 K when **o-1**·3CH_3_CN·H_2_O was initially heated, consistently with the low-spin electronic structures of the two LS-Co^III^(3,5-DTCat^2–^)(3,5-DTSQ^·–^) species. The magnetic susceptibility abruptly increased at approximately 350 K to 6.21 cm^3^ mol^−1^ K at 400 K, which is close to that of two HS-Co^II^(3,5-DTSQ^·–^)_2_ units. Magnetic data could not be collected above 400 K due to limitations associated with the maximum operating temperature of our magnetometer. The observed change in magnetic susceptibility suggests that the thermally induced LS-Co^III^(3,5-DTCat^2–^)(3,5-DTSQ^·–^) to HS-Co^II^(3,5-DTSQ^·–^)_2_ VT transition was almost complete, with *T*_c↑_ = 390 K (Supplementary Fig. S22), indicative of charge transfer from Cat^2–^ to the Co(III) center. **o-1**·3CH_3_CN·H_2_O displays a gradual incomplete transition from a high- to a low-spin state during backtracking, with *T*_c↓_ = 360 K, which is attributable to the loss of solvent molecules at high temperature [49]. This is consistent with the thermogravimetric data of **o-1**·3CH_3_CN·H_2_O, which shows the loss and partial loss of water and acetonitrile molecules (Supplementary Fig. S23). On the other hand, **c-1**·H_2_O exhibited different behavior (Supplementary Fig. S24). During initial heating, the magnetic susceptibility of **c-1**·H_2_O remained in the range consistently with slightly coupled LS-Co^III^(3,5-DTCat^2–^)(3,5-DTSQ^·–^) species below 300 K, with a more abrupt spin transition observed at approximately 300 K. A maximum value of 1.99 cm^3^ mol^−1^ K was observed at 325 K, which increased gradually with increasing temperature to 3.11 cm^3^ mol^−1^ K at 400 K. In addition, **c-1**·H_2_O exhibited a hysteresis loop of nearly 21 K at approximately 300 K (critical temperatures: *T*_c↑_ = 311 K, *T*_c↓_ = 290 K) upon cooling, which is rare among general VT systems [50–54]. These results suggest that **c-1**·H_2_O favors cooperativity more than **o-1**·3CH_3_CN·H_2_O; this higher cooperativity is possibly ascribable to the π-conjugated structure of **c-1**·H_2_O. The relatively high degree of conjugation in **c-1**·H_2_O compared with that in **o-1**·3CH_3_CN·H_2_O results in long-range interactions in the 1D chain in **c-1**·H_2_O. Moreover, short-range interactions occur between the 1D chains in the crystal packing frame. Consequently, intermolecular interactions are strong in **c-1**·H_2_O, which leads to the observed hysteresis loop in the magnetic susceptibility curve. Hence, we confirmed the effectiveness of the strategy adopted in this system to form polymers with switchable physical properties through the introduction of ligands that are photocyclizable. In addition, we used variable-temperature EPR spectroscopy to confirm the VT transition in **c-1**·H_2_O (Supplementary Fig. S25).

### Electrical conductivity studies

The current–voltage (*I*–*V* curves) characteristics of **o-1**·3CH_3_CN·H_2_O and **c-1**·H_2_O were examined to further explore the multifunctionalities of the VT complexes and the directionalities of the dual radicals (Supplementary Fig. S26). The *I*–*V* curves of **o-1**·3CH_3_CN·H_2_O and **c-1**·H_2_O exhibit almost linear relationships, indicative of low but different conducting performance. A two-orders-of-magnitude higher current was detected using **o-1**·3CH_3_CN·H_2_O compared with **c-1**·H_2_O, which exhibits insulating behavior. We then studied the temperature dependence of the electrical conductivity *σ* in **o-1**·3CH_3_CN·H_2_O (Fig. 6b). The results of three selected single crystals show crossover at approximately 330 K, where the VT transition occurs, which indicates that semi-conductivity is possibly synchronized with thermally induced valence tautomerism. The average conductivity at 327 K was determined to be 2.1 × 10^–8^ S cm^−1^, which is characteristic of semiconductors with very small numbers of free carriers (electrons in the conduction bands or holes in the valence bands) compared with the number of atoms. Here, we observed synergy between magnetism and conductivity in **o-1**·3CH_3_CN·H_2_O. Electrical conductivity increased with increasing temperature below the transition temperature, which is a characteristic semiconductor behavior. However, electrical conductivity was observed to decrease with increasing temperature above 330 K; hence, **o-1**·3CH_3_CN·H_2_O showed a behavior of decreasing conductivity with increasing temperature, which is ascribable to electron transfer during valence tautomerism. Similar behavior has also been observed in a TTF–chloranil charge-transfer salt, which exhibits a neutral (N) to ionic (I) phase transition [55]. However, such behavior was observed for the first time in a single-crystal VT complex in this study. Notably, while we evaluated the conductivity of the *ab* plane in a single crystal, different results were obtained regarding the *c*-axis. The resistivity of a single crystal decreased with increasing temperature when two Au wires were placed along the *c*-axis (Supplementary Fig. S27), consistently with classic semiconductor behavior. A slight increase was observed between 340 and 360 K, which is likely due to a crack in the crystal caused by the high temperature [56,57].

The conductivity in the *ab* plane is attributable to charge transfer from the ligand to the Co ion in the VT complex. The Co_1_ center in the 1D chain of **o-1**·3CH_3_CN·H_2_O is coordinated by the catecholate and semiquinone ligands. Directional charge transfer from the catecholate ligand to the Co_1_ ion (corresponding to Radical 1) occurred during heating, whereas the direction of electron transfer was random for Co_2_ (corresponding to Radical 2) because of the averaged dioxolene ligands. Radicals 1 and 2 are distributed in the *ab* plane; hence, all electron-transfer processes occur in the *ab* plane, i.e. directional or non-directional electron hopping capable of forming short-range conductive pathways occurs in the *ab* plane (Supplementary Fig. 6c). However, no charge transfer between the ligand and the metal was observed along the *c*-axis, as this corresponds to the direction of the 6F-DAE-py_2_ ligand.

To determine the origin of the anomalous semiconductive behavior in **o-1**·3CH_3_CN·H_2_O, the electrical conductivity was further analysed. Several single crystals were subjected to direct current (DC) electrical resistivity studies in the range of 200–360 K. The low conductivity and strong thermal activation observed below 300 K indicate that charge transport occurred by polaron hopping. In terms of structure, intra/intermolecular packing may provide a likely electronic pathway along the *c*-axis within **o-1**·3CH_3_CN·H_2_O, as shown in Supplementary Fig. S28. The Arrhenius equation was used to fit the linear region of the curve: $lo{g}_{10}( \rho ) = lo{g}_{10}({\rho }_0) + ( {{\raise0.7ex\hbox{${k{E}_a}$} \!\mathord{/ {\vphantom {{k{E}_a} {lo{g}_{10}e}}}} \!\lower0.7ex\hbox{${lo{g}_{10}e}$}}} ) \cdot ( {{\raise0.7ex\hbox{$1$} \!\mathord{/ {\vphantom {1 T}}} \!\lower0.7ex\hbox{$T$}}} )$. The activation energies of **o-1**·3CH_3_CN·H_2_O at various temperatures are shown in Supplementary Table S9 along with the corresponding crossover temperatures. The observed increase in conductivity is likely due to a change in activation energy caused by a dynamic charge fluctuation ascribable to charge transfer. According to the Arrhenius plot, it has a lower activation energy (*E_a_*) in the relatively high temperature range (248−273 meV between 283 and 321 K) compared with the higher activation energy in the low temperature range (403−448 meV between 246 and 297 K). As a result, semiconductor behavior was observed before the VT transition.

### DFT calculations

The change (conducting anomaly) in conductivity observed in **o-1**·3CH_3_CN·H_2_O upon valence tautomerism is attributable to a change in the rate of charge-carrier hopping. DFT calculations were used to reveal the synergistic mechanism responsible for the conductivity and magnetism observed in **o-1**·3CH_3_CN·H_2_O (see Supplementary information for further details). The B3LYP (for isolated systems) and PBE + U (where the *U* value of the Co *d* electrons in extended systems is set to 6 eV) functionals were used to describe the different spin states in the low- and high-temperature structures (Supplementary Fig. S31). The abovementioned DFT methods correctly reproduced the electronic structures of the CoL_2_ units (where L represents the doubly charged 3,5-DTCat^2–^, singly charged 3,5-DTSQ^·–^ or neutral chelating 3,5-di-tert-butyl-o-benzoquinone ligand), which is consistent with the experimental magnetic susceptibility results. The CoL_2_ units are in the low-spin LS-Co^III^(3,5-DTCat^2–^)(3,5-DTSQ^·–^) state at low temperatures, whereas they are in the high-spin HS-Co^II^(3,5-DTSQ^·–^)_FM_(3,5-DTSQ^·–^)_FM_ state at high temperatures.

The PBE + U band structure of **o-1**·3CH_3_CN·H_2_O reveals that the system should exhibit high conductivity due to the small band gap (Supplementary Fig. S30); however, this prediction is inconsistent with the experimental results. Pure DFT functionals are known to underestimate band gaps. In principle, the DFT + U formalism can help counteract this [58]; however, while effective on-site coulombic repulsions are only added to the *d* electrons of the Co atoms, the bands close to the Fermi level mainly comprise the frontier orbitals of the organic ligands. In principle, the over-delocalization problem can be resolved using a hybrid functional (e.g. B3LYP, PBE0) to predict a more accurate band gap; however, this requires calculating the exact exchange term, which entails a high cost under periodic boundary conditions in the standard implementation and is consequently almost infeasible for such a large system.

According to the band structures and calculations of the isolated systems, the bare on-site coulombic repulsion associated with the CoL_2_ units is much larger than the bandwidths formed by orbital interactions between CoL_2_ units. Therefore, the system is likely to be in a spin-localized state as a Mott insulator. Furthermore, a band structure only reflects conductivity when electrons are added or removed from bands in the vicinity of the Fermi level without electron configuration and lattice relaxation. In addition, the hopping mechanism should dominate conductivity in the case of **o-1**·3CH_3_CN·H_2_O according to the temperature-dependent electrical conductivity data; hence, the charged CoL_2_ units relax to different configurations with lower energies during charge transport.

**Figure 7.**
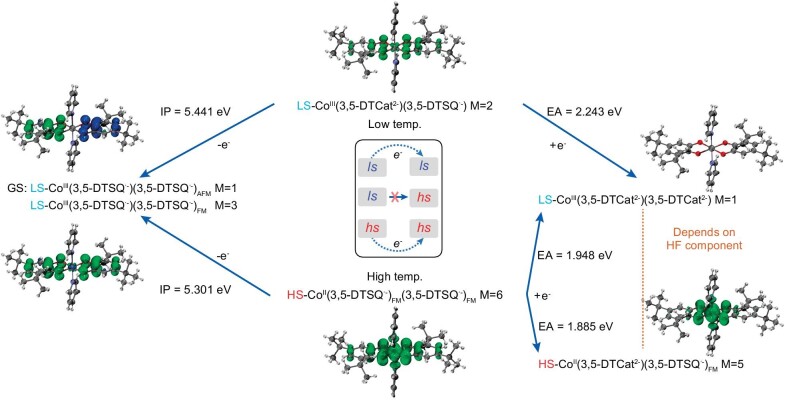
B3LYP KS-DFT-calculated stable electronic configurations and the corresponding spin-density isosurfaces of the variously charged CoL_2_ units of **c-1**·H_2_O at low (100 K) and high (380 K) temperatures. The system may converge to the V^−^ configuration with an HS-Co^II^ center and FM-coupled semiquinonate ligand. Here, PBE, TPSSh, and B3LYP predicted the I^−^ configuration, whereas PBE0, with a more exact exchange admixture, predicted the V^−^ configuration. Color code: Co, dark violet; O, red; N, blue; C, gray; H, white. Inset: schematic diagram of the electron-hopping event, which occurs only between CoL_2_ units with the same spin configuration.

Based on the molecular structures of the CoL_2_ unit at low (100 K) and high (380 K) temperatures extracted from **o-1**·3CH_3_CN·H_2_O, KS wave functions were calculated using DFT functionals, starting with the listed initial guesses (Supplementary Table S12) and selected using the SCF procedure, the results of which are summarized in Fig. 7. For [CoL_2_]^+^, which is positively charged, the system always converges to the LS-Co^III^(3,5-DTSQ^·–^)(3,5-DTSQ^·–^)_AFM_ (II^+^) (AFM = antiferromagnetic) configuration as the ground state with a closed-shell LS-Co^III^ center and a pair of AFM-coupled semiquinonate radicals. Hence, the spin state of Co should convert from HS-Co^II^ to LS-Co^III^, even at high temperatures. The two semiquinonate ligands can also be FM-coupled (FM = ferromagnetic), albeit with slightly higher energy. B3LYP KS-DFT calculations reveal that the low- and high-temperature molecular structures differ by 0.0158 and 0.0052 eV, respectively. For negatively charged [CoL_2_]^−^, a system with a low-temperature molecular structure is likely to converge to the closed-shell LS-Co^III^(3,5-DTCat^2–^)(3,5-DTCat^2–^) (I^−^) configuration. However, the high-temperature molecular structure is divided in a manner that depends on the exact exchange ratio, where the I^−^ configurations and the open-shell HS-Co^II^(3,5-DTCat^2–^)(3,5-DTSQ^·–^)_FM_ (V^−^) configurations are close in energy (Supplementary Tables S13–S16).

The electron-transfer matrix elements were calculated based on these electronic configurations and the pairwise spatial relationships of the CoL_2_ units in the two crystals. According to these calculations, **o-1**·3CH_3_CN·H_2_O displays 2D semiconducting properties in the *ab* plane, with the symmetric CoL_2_ layers (Co_2_) playing major conductivity roles, consistently with anisotropic conductivity results (Fig. 6b and Supplementary Fig. S27). Electrons act as carriers at high temperature; however, hopping events are dominated by hole conduction at low temperatures.

In the case of **o-1**·3CH_3_CN·H_2_O, the carrier density increases with increasing temperature due to thermal excitation, which is common for a semiconductor; consequently, the conductivity increases. The spin states of the CoL_2_ units begin to undergo low- to high-spin conversion at approximately 300 K; as a result, a CoL_2_ unit may be surrounded by other units with different spin states. The rate of electron transfer then decreases to zero, leading to a decline in conductivity, which explains the peak conductivity observations (Fig. 6b). Therefore, for the electrical conductivity study, the Arrhenius analysis determined the origin of the semiconductive behavior before 320 K, while DFT calculations helped illustrate the mechanism of the unusual change in electrical conductivity during the VT transition process.

## CONCLUSION

The photoisomerizable 6F-DAE-py_2_ ligand was used to synthesize two VT CPs, namely **o-1**·3CH_3_CN·H_2_O and **c-1**·H_2_O, which display VT and photoconversion behavior. Two crystallographically independent SQ^·–^ radicals arranged along the 1D chain were observed in **o-1**·3CH_3_CN·H_2_O using SC-XRD. Therefore, two clearly distinct radical signals were observed in the high-field/-frequency single-crystal EPR spectra of a VT complex for the first time. The directional variations of these dual radicals, as well as their 180° periods, were then confirmed by using angle-dependent EPR spectroscopy. Furthermore, **o-1**·3CH_3_CN·H_2_O exhibited anisotropic conductivity/magnetism synergy. A conductive anomaly close to the VT transition temperature was observed along the *ab* plane in **o-1**·3CH_3_CN·H_2_O, while insulator-like behavior was observed along the *c*-axis. Meanwhile, the synergistic mechanism was explained using DFT calculations. This study is of considerable significance with regard to the anisotropy and synthesis of multifunctional bistable magnetic materials in the molecular magnetism field.

## EXPERIMENTAL PROCEDURES

### Resource availability

#### Lead contact

Further information and requests for resources should be directed to and will be fulfilled by the lead contact, Masahiro Yamashita (yamasita@agnus.chem.tohoku.ac.jp), and Zhao-Yang Li (zhaoyang@nankai.edu.cn).

#### Materials availability

Complex **o-1**·3CH_3_CN·H_2_O and **c-1**·H_2_O can be produced following the procedures outlined below from standard reagents and procedures.

#### Data and code availability

Crystal data for **o-1**·3CH_3_CN·H_2_O and **c-1**·H_2_O are available from the Cambridge Crystallographic Data Centre under CCDC: 2175512, 2175513. Computational output is available on request.

### Synthesis

All reagents were obtained from commercial sources and used without further treatment. The open/closed-form ligands **o-L** and **c-L** were synthesized according to previously reported procedures (Supplementary Scheme S1) [59].

The open-form 6F-DAE-py_2_ ligand (**o-L**) was synthesized as previously described [59]. Based on our experience, we note that separation and purification by column chromatography is very time-consuming; therefore, a gradated mixture of dichloromethane and ethyl acetate (from 5 : 1 to 1 : 1) was used as the eluent to enhance separation efficiency. A single crystal of the closed-form 6F-DAE-py_2_ ligand (**c-L**) was isolated from an acetonitrile solution of **o-L** by irradiation with UV light (365 nm) for two hours.

**o-1**·3CH_3_CN·H_2_O was successfully obtained as deep-green hexagonal crystals by the layer-by-layer diffusion reaction of cobalt(II) acetate (4.98 mg, 0.02 mmol) in H_2_O (1 mL), H_2_Cat (9.34 mg, 0.042 mmol) in MeCN (1 mL) and 6F-DAE-py_2_ (10.44 mg, 0.02 mmol) in MeCN (1 mL). Yield: 45%. Anal. calcd.: C 62.28, H 5.52, N 2.74%. Found: C 62.246, H 5.784, N 2.892%. IR (KBr pellets, cm^–1^): 1540, 1505, 1480, 1440, 1421, 1375, 1354, 1277, 1245, 1215. The closed form of metal complex **c-1**·H_2_O was prepared in two ways. Method 1: Single crystals of **c-1**·H_2_O were obtained from a DMF solution of **o-1**·3CH_3_CN·H_2_O upon irradiation with 365-nm UV light. Single crystals of **o-1**·3CH_3_CN·H_2_O (8 mg) were dissolved in DMF (1.5 mL) in an NMR tube and then layered with acetonitrile after 2 h of irradiation with 365-nm UV light. Single crystals suitable for SC-XRD were collected after placing the NMR tube in a dark room for 2 h. Method 2: A solution of **o-L** (15.66 mg, 0.03 mmol) in acetonitrile (2 mL) was irradiated by 365-nm UV light to yield a solution of **c-L**, which was then reacted with cobalt(II) acetate (7.47 mg, 0.03 mmol) in water (1 mL) and H_2_Cat (14.23 mg, 0.064 mmol) in MeCN (1 mL) using the layer-by-layer technique. Single crystals of irregularly shaped **c-1**·H_2_O were collected in 23% yield after storage for 3 days in a dark room. IR (KBr pellets, cm^–1^): 1591, 1567, 1547, 1534, 1514, 1485, 1476, 1448, 1426.

### X-ray crystallography

**o-1**·3CH_3_CN·H_2_O and **c-1**·H_2_O were subjected to single-crystal X-ray diffractometry on a Rigaku XtalAB PRO MM007 DW diffractometer with Mo*K_α_* (*λ* = 0.71073 Å) radiation at 293 and 100 K, respectively. All collected data were integrated and revived using CrystalAlice Pro software. All structures were solved with the Olex2 structure-solution program using direct methods and refined by full-matrix least-squares against *F^2^* in the SHELX refinement package. All non-hydrogen atoms were refined anisotropically. Free solvent molecules were removed using the squeeze command. A high beamstop theta(min) limit set and ignorance of the very high angle data that is inappropriate for highly disordered structures account for the relatively high *R*_1_ values. CCDC 2175512 (**o-1**·3CH_3_CN·H_2_O) and 2175513 (**c-1**·H_2_O) contain supplementary crystallographic data for compounds **o-1**·3CH_3_CN·H_2_O and **c-1**·H_2_O. These data can be obtained free of charge from the Cambridge Crystallographic Data Centre at www.ccdc.cam.ac.uk/data _request/cif.

### NMR spectroscopy

NMR spectra were recorded at field strengths of 7.9 T (400 MHz, Bruker Avance II instrument equipped with a broad band fluorine observation (BBFO) probe) and 14.09 T (600 MHz, Bruker Avance III instrument equipped with a QNP Cryoprobe). The temperature-dependent residual non-deuterated methanol signal in deuterated methanol was used to calibrate the temperature [60]. ^1^H NMR spectra were referenced against the residual proton signal in DMF-d_7_ [61]. The deuterated solvent (DMF-d_7_, Sigma–Aldrich) was dried and degassed using conventional methods prior to use. The NMR samples were prepared and stored in an inert atmosphere in tubes with Teflon plugs (J. Young valves).

NMR DFT calculations were performed using the Gaussian 09 (revision D.01) program. Theoretical studies on VT complexes have shown that the OPBE [62] and B3LYP^*^ [63] functionals give reliable results [64]. More recently, DFT calculations on the VT [Co(3,5-DTSQ^·–^)(3,5-DTCat^2–^)(4-papy)_2_] complex have been reported using the OPBE functional [22]. For this reason, the unrestricted B3LYP^*^ functional and the def2svp [65] basis set were used to optimize the geometries of model complexes of the VT 1D chain. A quadratically convergent self-consistent field procedure was applied when convergence could not be achieved using the standard first-order procedure. A ‘superfine’ integration grid was used and symmetries were not restricted during optimization. In each case, the absence of an imaginary frequency confirms that a local minimum is located on the potential energy surface. Broken-symmetry calculations were performed with the UB3LYP^*^ functional and the def2tzvp [65] basis set using the ‘scf = xqc’ procedure and ‘superfine’ integration grids; in these calculations, the stabilities of the wave functions were tested [66] and, where necessary, reoptimized. The *xyz* coordinates of the optimized structures, energies from single-point calculations and relevant spin-density maps are provided herein.

### Magnetization

Magnetization experiments were carried out using a Quantum Design MPMS 5S SQUID magnetometer equipped with a 5-T magnet. Magnetization (*M*) was measured as the temperature was increased from 5 to 400 K at 5 K·min^−1^ in a 5000-Oe field (*H*) in three heating and cooling cycles. The magnetic susceptibility per mole (*χ_m_*) was calculated as *χ_m_* = *M*_m/_*H*.

### Electrical conductivity

A single hexagonal crystal of **o-1**·3CH_3_CN·H_2_O was measured along the *ab* plane and the *c*-axis by using the two-probe DC method (Keithley 2400) using silver paste electrodes and soft gold-coated spider silk fibers with a diameter of approximately 25 μm as electrical wires. The proper orientation was established through the face index of the crystal by means of X-ray diffraction. The temperature-dependent conductivity was measured within a sealed cycle cryostat in the temperature range of 150−360 K.

### Other characterizations

Powder XRD was performed on a Rigaku charge-coupled device (CCD) diffractometer. Electronic spectroscopy was performed on a Shimadzu UV3600IPLUS ultraviolet/visible/near-infrared spectrophotometer. EPR spectroscopy was performed on a Bruker EMX Plus spectrometer operating in the X-band (9.1 GHz) and Q-band (34.0 GHz) frequencies. High-field (∼13.3 T) and high-frequency (∼360 GHz) single-crystal EPR spectroscopy was performed on a terahertz electron spin resonance apparatus. EPR spectra were acquired at selected temperatures with variations in temperature measured using a helium continuous-flow thermostat. Angle-dependent EPR spectra were obtained by rotating a single crystal of **o-1**·3CH_3_CN·H_2_O in the plane perpendicular to the *ab* plane. Electrical conductivity was measured using a helium flow thermostat and temperature-dependent conductivity was determined with a voltage bias of 1.5 V at a ramping rate of 1 K min^−1^. A single crystal of **o-1**·3CH_3_CN·H_2_O fixed on a quartz tube was photographed on a Rigaku XtalAB PRO MM007 DW diffractometer at room temperature. Face indexing was carried out by using the CrysAlisPro 171.40.84a program.

## Supplementary Material

nwad047_Supplemental_Files
