# Supplementary material for: Dual-radical-based molecular anisotropy and synergy effect of semi-conductivity and valence tautomerization in a photoswitchable coordination polymer
Source: Natl Sci Rev. 2023 Feb 23;10(6):nwad047. doi: 10.1093/nsr/nwad047 (PMC10354699; doi:10.1093/nsr/nwad047)
Supplement: nwad047_Supplemental_Files [file nwad047_supplemental_files.zip › Revised SI-NSR-20230221.pdf]

Supplementary Information

Dual-radical-based molecular anisotropy and synergy effect of semi-conductivity and valence tautomerization in a photoswitchable coordination polymer

Jing-Wei Dai,[4,†] Yu-Qin Li,[1,†] Zhao-Yang Li,[1,*] Hai-Tao Zhang,[2] Carmen Herrmann,[2] Shohei Kumagai,[3] Marko Damjanović,[5] Markus Enders,[5] Hiroyuki Nojiri,[6] Masakazu Morimoto,[7] Norihisa Hoshino,[8] Tomoyuki Akutagawa,[8] and Masahiro Yamashita[3,‡,**]

[1]School of Material Science and Engineering, Nankai University, Haihe Educational Park, Tianjin 300350, China

[2]Institute of Inorganic and Applied Chemistry, University of Hamburg, Hamburg 22761, Germany

[3]Department of Chemistry, Graduate School of Science, Tohoku University, Sendai 980-8578, Japan

[4]State Key Laboratory of Medicinal Chemical Biology, Nankai University, Tianjin 300071, China

[5]Institute of Inorganic Chemistry, University of Heidelberg, Heidelberg D-69120, Germany

[6]Institute for Materials Research, Tohoku University, Sendai 980-8577, Japan

[7]Department of Chemistry, Rikkyo University, Tokyo 171-8501, Japan

[8]Institute of Multidisciplinary Research for Advanced Materials (IMRAM), Tohoku University, Sendai 980-8577, Japan

[†]These authors contributed equally

[‡]Lead contact

[*]Correspondence: zhaoyang@nankai.edu.cn

[**]Correspondence: yamasita@agnus.chem.tohoku.ac.jp

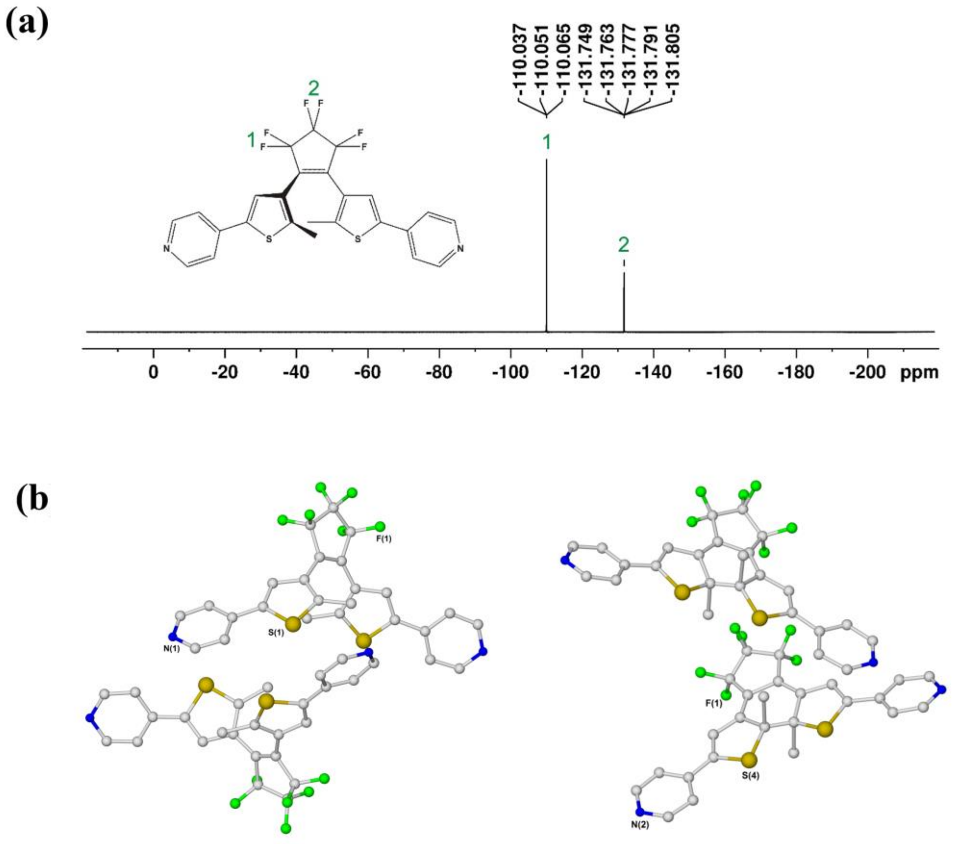

Figure S1. (a) Assigned ^{19}F NMR spectrum of **o-L** in $CDCl_3$ at 295.0 K recorded at 14.09 T. (b) Single-crystal structures of **o-L** (left) and **c-L** (right) crystallized from acetonitrile/hexane and ethyl acetate/hexane by the vapor-diffusion method. Color code: N, blue; C, gray; S, yellow; F, green.

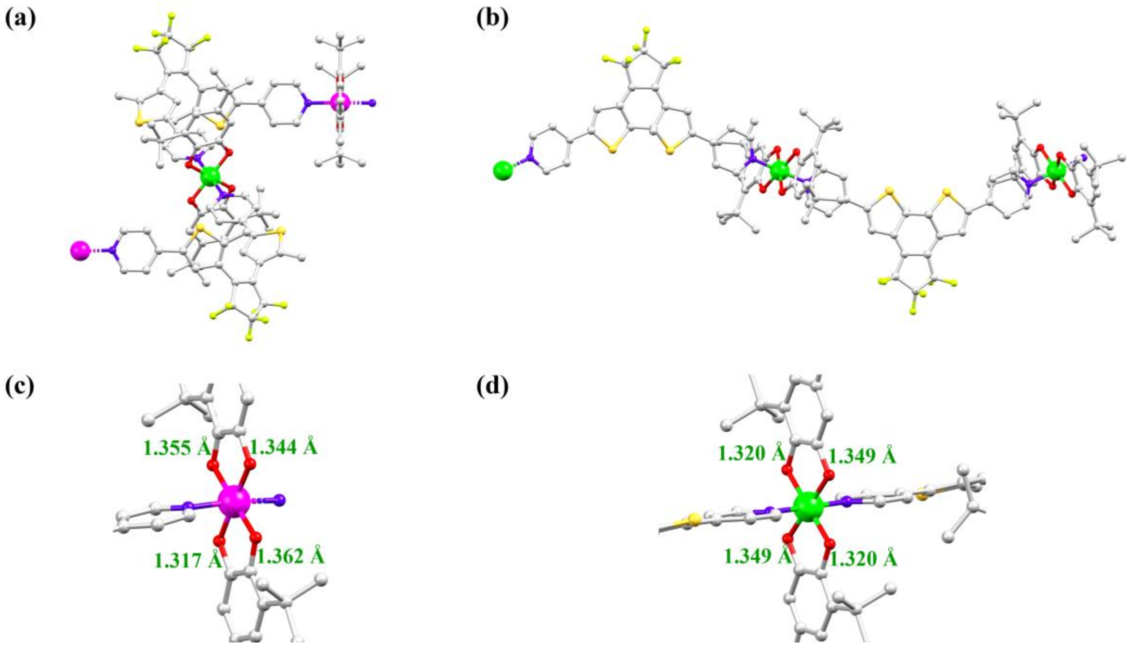

Figure S2. Asymmetric units of (a) **o-1**·3CH_3CN·H_2O and (b) **c-1** H_2O and the C−O bond lengths of the (c) Co_1 and (d) Co_2 units. Hydrogen atoms and solvent molecules are omitted for clarity. Color code: Co_1, pink; Co_2, green; O, red; N, blue; C, gray; S, orange; F, yellow–green.

Table S1. Crystallographic data for **o-1**·3CH_3CN·H_2O and **c-1** H_2O at 100 K

Complex	**o-1**·3CH_3CN·H_2O	**c-1**·H_2O
Formula	$C_{59}H_{67}CoN_5O_5S_2F_6$	$C_{53}H_{58}CoN_2O_5S_2F_6$
Molecular weight	1163.21	1040.06
Temperature/K	100.15	100.15
Radiation	MoKα (λ = 0.71073)	MoKα (λ = 0.71073)
Crystal system	hexagonal	monoclinic
Space group	$P6_3/m$	$P2_1/n$
a/Å	25.600(3)	15.822(3)

Complex	**o-1**·$3CH_3CN·H_2O$	**c-1**·H_2O
b/Å	25.600(3)	11.732(2)
c/Å	31.061(6)	33.692(7)
α/°	90	90
β/°	90	103.15(3)
γ/°	120	90
V/Å^3	17629(5)	6090(2)
Z	12	4
ρ_{calc}/g cm^{-3}	1.155	1.134
μ/mm^{-1}	0.422	0.409
F(000)	6396.0	2172.0
2θ range/°	8.012 to 50.052	6.06 to 46.51
Reflections collected	33263	17234
R_{int}	0.0460	0.0452
GOF on F^2	1.077	1.032
Final R indexes [I >= 2σ (I)]	R_1 = 0.1066, wR_2 = 0.2732	R_1 = 0.1000, wR_2 = 0.2377
Final R indexes [all data]	R_1 = 0.1502, wR_2 = 0.3163	R_1 = 0.1289, wR_2 = 0.2592
CCDC number	2175512	2175513

Table S2. Bond-length data for **o-1**·$3CH_3CN·H_2O$ at 100 K

**Atoms**	**Lengths/Å**	**Atoms**	**Lengths/Å**
Co1−O2	1.870(7)	C33−C34	1.391(8)

Co1−O4	1.923(9)	C33−C36	1.394(9)
Co1−O1	1.886(9)	C1−C6	1.441(13)
Co1−O3	1.895(8)	C45−C50	1.62(4)
Co1−N1	1.930(5)	C25−C24	1.519(12)
Co1−N1[1]	1.930(5)	C25−C26	1.541(12)
Co2−O6[2]	1.890(5)	C39−C38	1.403(11)
Co2−O6	1.890(5)	C39−C40	1.400(11)
Co2−O5[2]	1.890(5)	C50−C51	1.554(15)
Co2−O5	1.890(5)	C50−C9	1.612(16)
Co2−N2[2]	1.950(5)	C50−C65	1.43(5)
Co2−N2	1.950(5)	C37−C36	1.363(9)
S2−C32	1.727(6)	C38−C43	1.409(11)
S2−C29	1.719(6)	C4−C5	1.388(14)
S1−C18	1.737(6)	C4−C44	1.580(15)
S1−C21	1.726(6)	C6−C5	1.380(14)
O2−C2	1.356(11)	C6−C47	1.482(14)
O4−C8	1.316(9)	C40−C41	1.363(11)
O1−C1	1.342(13)	C43−C56	1.535(13)
F5−C26	1.353(10)	C43−C42	1.415(14)
O3−C7	1.361(9)	C47−C54	1.61(4)
N1−C15	1.343(8)	C47−C48	1.568(18)
N1−C13	1.348(7)	C47−C67	1.48(4)
O6−C39	1.319(8)	C58−C56	1.553(10)

F2−C24	1.346(11)	C44−C52	1.63(6)
O5−C38	1.348(10)	C44−C46	1.49(3)
F1−C24	1.355(11)	C44−C66	1.41(7)
F6−C26	1.343(9)	C56−C57	1.568(12)

Table S3. Bond-length data for **c-1** H_2O at 100 K

**Atoms**	**Lengths/Å**	**Atoms**	**Length/Å**
C1−C2	1.397(10)	C36−C37	1.349(10)
C1−C8	1.423(10)	C36−C47	1.542(11)
C1−O1	1.366(8)	C37−C38	1.471(10)
C2−C5	1.421(9)	C37−C41	1.460(9)
C2−O2	1.329(8)	C38−C39	1.529(11)
C3−C4	1.409(11)	C38−F1	1.348(8)
C3−C20	1.395(10)	C38−F2	1.355(8)
C3−O3	1.353(9)	C39−C40	1.500(12)
C4−C17	1.452(12)	C39−F3	1.449(12)
C4−O4	1.340(8)	C39−F4	1.328(10)
C5−C6	1.384(10)	C40−C41	1.499(11)
C6−C7	1.388(10)	C40−F5	1.432(9)
C6−C9	1.556(10)	C40−F6	1.279(9)
C7−C8	1.409(10)	C41−C42	1.349(10)
C8−C13	1.498(11)	C42−C43	1.440(9)
C9−C10	1.486(13)	C42−C45	1.559(12)

C9−C11	1.535(12)	C43−C44	1.349(10)
C9−C12	1.523(12)	C44−C49	1.472(9)
C13−C14	1.576(13)	C44−S2	1.783(8)
C13−C15	1.583(14)	C45−C46	1.657(11)
C13−C16	1.572(12)	C45−C47	1.432(12)
C17−C18	1.386(12)	C45−S2	1.839(10)
C17−C25	1.521(14)	C47−C48	1.657(11)
C18−C19	1.435(12)	C47−S1	1.843(8)
C19−C20	1.373(12)	C49−C50	1.415(11)
C19−C21	1.530(12)	C49−C53	1.386(10)
C21−C22	1.522(16)	C50−C51	1.393(10)
C21−C23	1.578(14)	C51−N2	1.334(9)
C21−C24	1.501(14)	C52−C53	1.394(10)

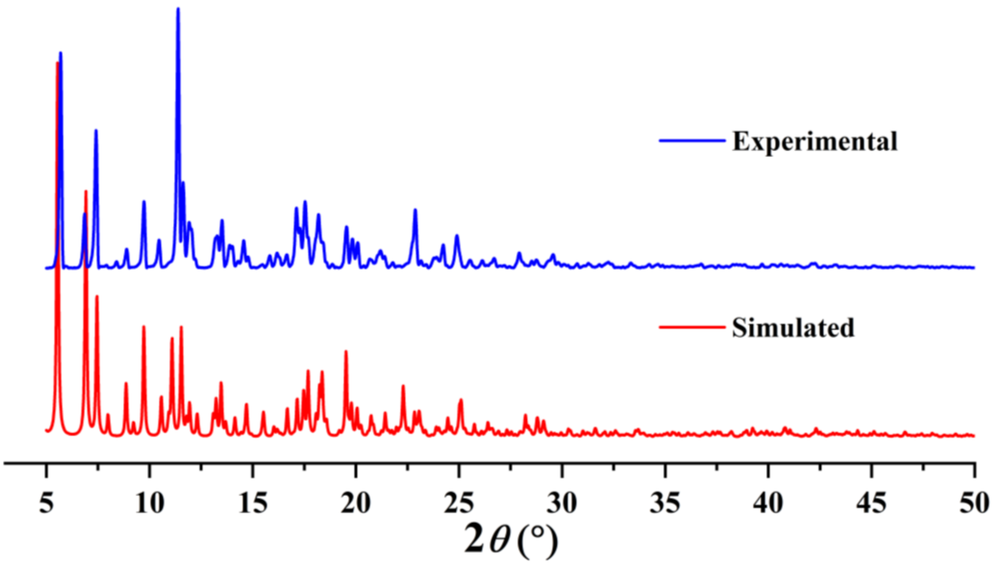

Figure S3. Experimental and simulated powder X-ray diffraction (XRD) patterns of **o-1**·$3CH_3CN·H_2O$ at room temperature.

The crystal structure of **o-1**·$3CH_3CN·H_2O$ reveals the presence of two types of CoL_2 units: an asymmetric unit (Co_1) and a symmetric unit (Co_2). The two ligands (L) are not crystallographically equivalent in the asymmetric CoL_2 unit (Co_1), whereas the symmetric CoL_2 unit (Co_2) is centrosymmetric with two equivalent ligands. Each type of CoL_2 unit forms a layer in the *ab* plane, resulting in both asymmetric and symmetric layers. The asymmetric CoL_2 units form a hexagonal lattice (three complexes as lattice points); consequently, three different pairwise relationships, with distances of 12.412 Å (type 1), 15.061 Å (type 2), and 17.084 Å (type 3), exist around CoL_2 (Figure S4); these are abbreviated as Co_1–Co_1-t1/t2/t3. The symmetric CoL_2 layer exhibits standard Kagome symmetry; therefore, all CoL_2 units in this layer are equidistant at 12.756 Å (Figure S5) and are abbreviated as Co_2–Co_2. Three types of interlayer pairs of CoL_2 units exist between the asymmetric and symmetric layers, with distances of 13.026 Å (type 1), 9.746 Å (type 2), and 14.219 Å (type 3) (Figure S6); these are abbreviated as Co_1–Co_2-t1/t2/t3. It is worth noting that the two CoL_2 units are linked by open-form 6F-DAE-py_2 in type 1.

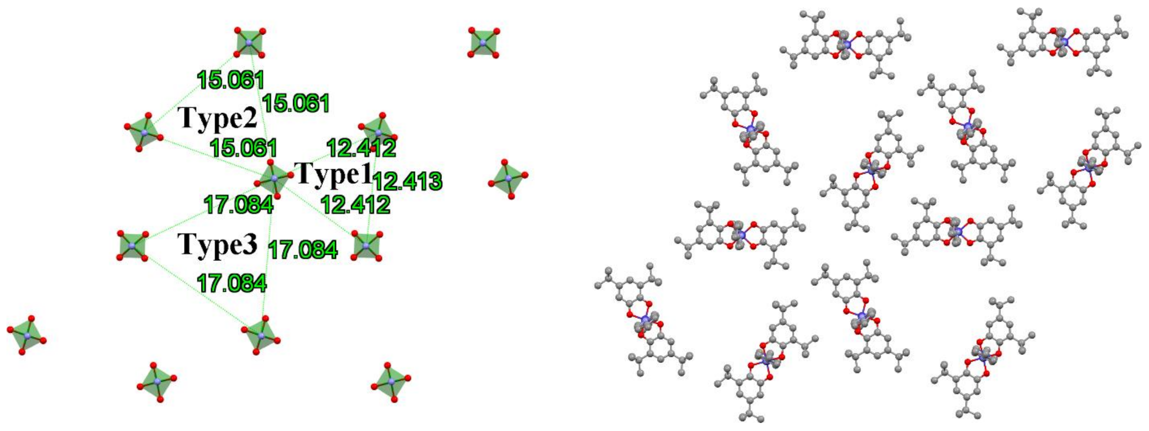

Figure S4. Octahedral representation (left) and ball-and-stick model (right) showing the CoL_2 units in the asymmetric layer and the three types of pairwise relationships (Co_1–Co_1-t1/t2/t3)

within the *ab* plane in **o-1**·3CH_3CN·H_2O at 100 K. Color code: Co, dark violet; O, red; N, blue; C, gray.

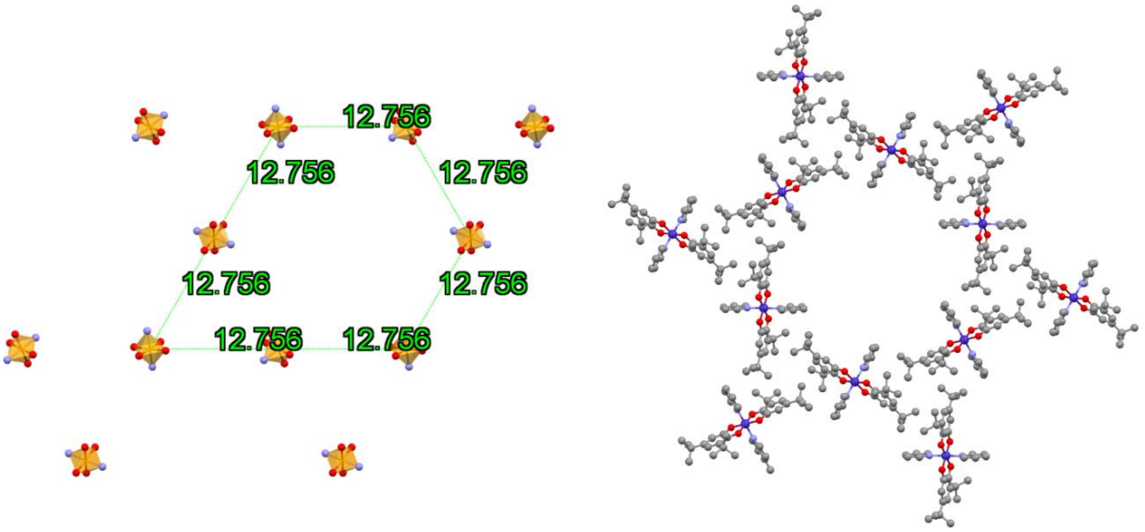

Figure S5. Octahedral representation (left) and ball-and-stick model (right) showing the CoL_2 units in the symmetric layer and the pairwise relationships (Co_2–Co_2) within the *ab* plane in **o-1**·3CH_3CN·H_2O at 100 K. Color code: Co, dark violet; O, red; N, blue; C, gray.

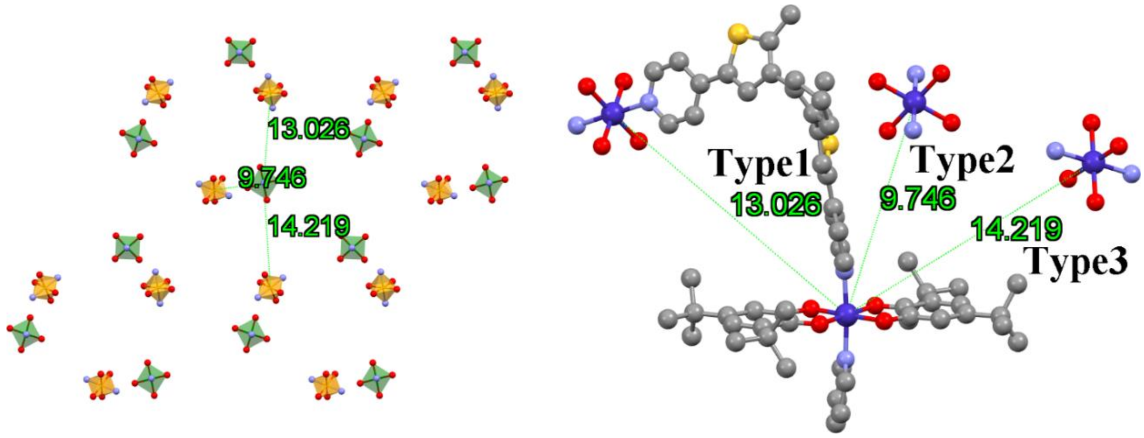

Figure S6. Octahedral representation (left) and ball-and-stick model (right) showing the CoL_2 units in the symmetric and asymmetric layers and the three types of interlayer CoL_2 pairs (Co_1–Co_2-t1/t2/t3) in **o-1**·3CH_3CN·H_2O at 100 K. Color code: Co, dark violet; O, red; N, blue; C, gray; S, yellow.

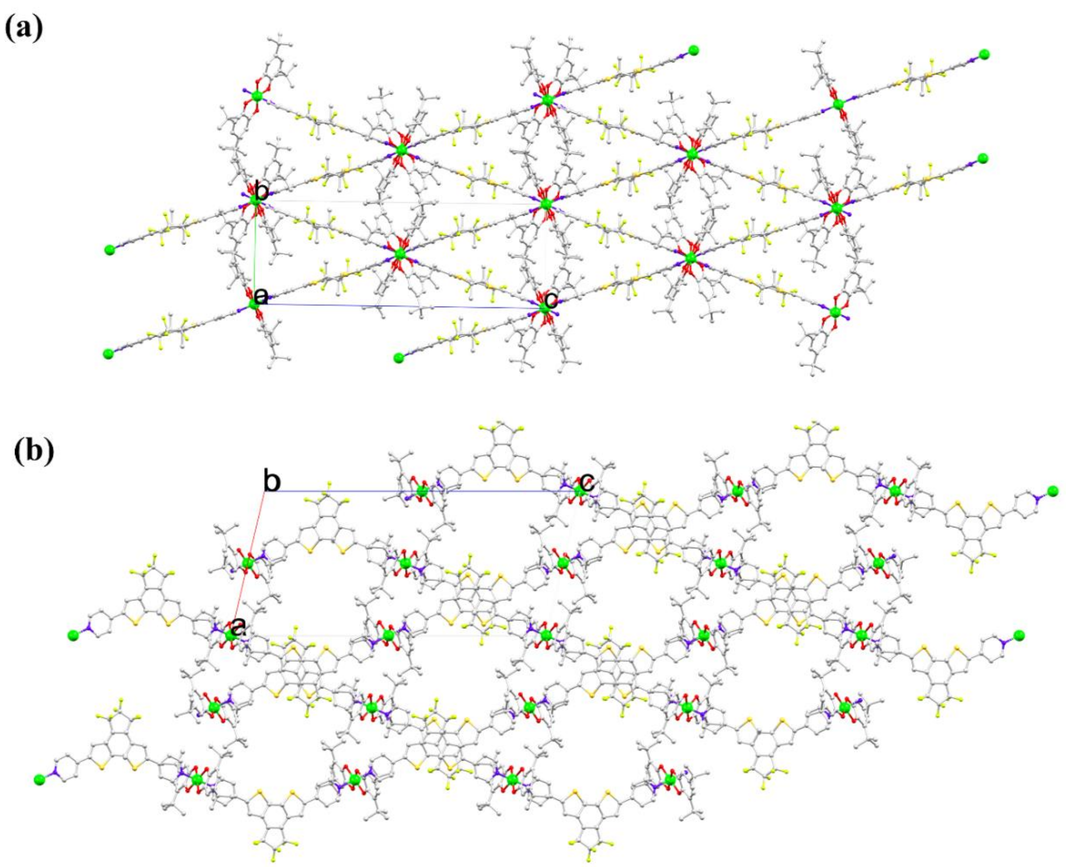

Figure S7. Packing diagrams for **c-1**·H_2O viewed from the (a) *a* and (b) *b* axes. Hydrogen atoms and solvent molecules are omitted for clarity. Color code: Co, green; O, red; N, blue; C, gray; S, orange; F, yellow–green.

Nuclear magnetic resonance (NMR) section

o-1·$3CH_3CN·H_2O$ is too insoluble in acetonitrile-d_3, toluene-d_8, and benzene-d_6 to enable NMR spectra to be acquired. An internal standard containing both hydrogen and fluorine atoms (*o*-$C_6H_4F_2$) was used to correlate the ^{1}H NMR resonances of **o-1**·$3CH_3CN·H_2O$ with its ^{19}F resonances. A comparison of the intensities of the ^{1}H and ^{19}F signals of **o-1**·$3CH_3CN·H_2O$ to those of the internal standard confirmed that the observed ^{19}F signals correspond to **o-1**·$3CH_3CN·H_2O$. Signals were assigned based on calculated spin densities and signal

intensities due to paramagnetism associated with the Co(III) ions. Variable-temperature experiments were performed to aid the assignment process.

The ^{1}H NMR spectrum of **o-1**·$3CH_3CN·H_2O$ in DMF-d_7 recorded at 340 K (Figure S8) and the corresponding variable temperature spectra (Figure S9–S11) are provided here. Eye guidelines are provided in the figure to highlight the valence-tautomeric transition process. Extensive heating at 420 K resulted in the gradual decomposition of **o-1**·$3CH_3CN·H_2O$. A comparison of the expanded region (+10 to −2 ppm) with the spectra of the free ligands reveals the absence of considerable amounts of free ligand in the solution of **o-1**·$3CH_3CN·H_2O$ (Figure S11). Hence, we confirmed that the one-dimensional chain of **o-1**·$3CH_3CN·H_2O$ is intact in solution.

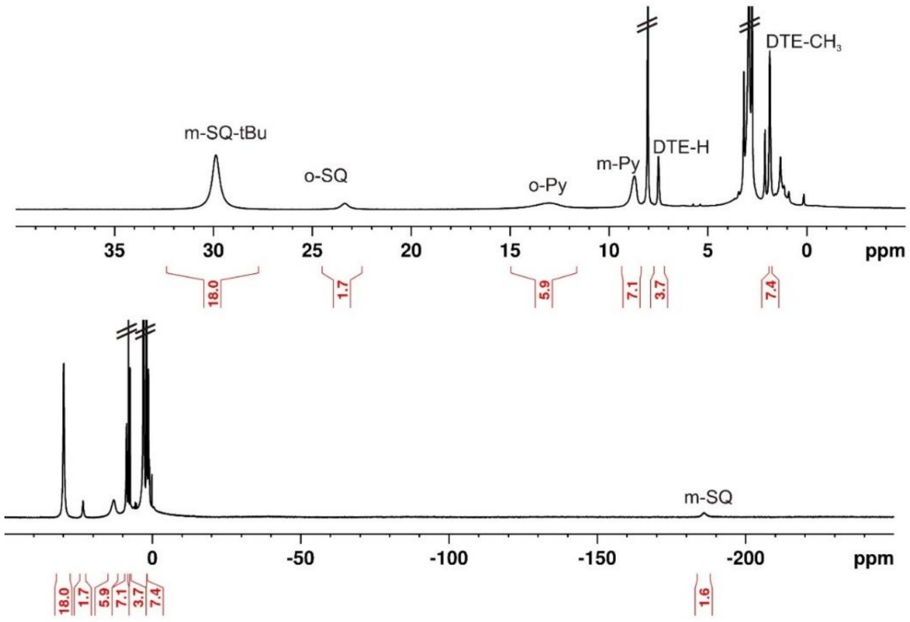

Figure S8. Assigned ^{1}H NMR spectrum of **o-1**·$3CH_3CN·H_2O$ in DMF-d_7 recorded at 340 K. Upper: expanded region (+40 to −5 ppm). The signal of the o-SQ-tBu group lies under the residual solvent signal (~3 ppm; see spectrum recorded at 420.0 K).

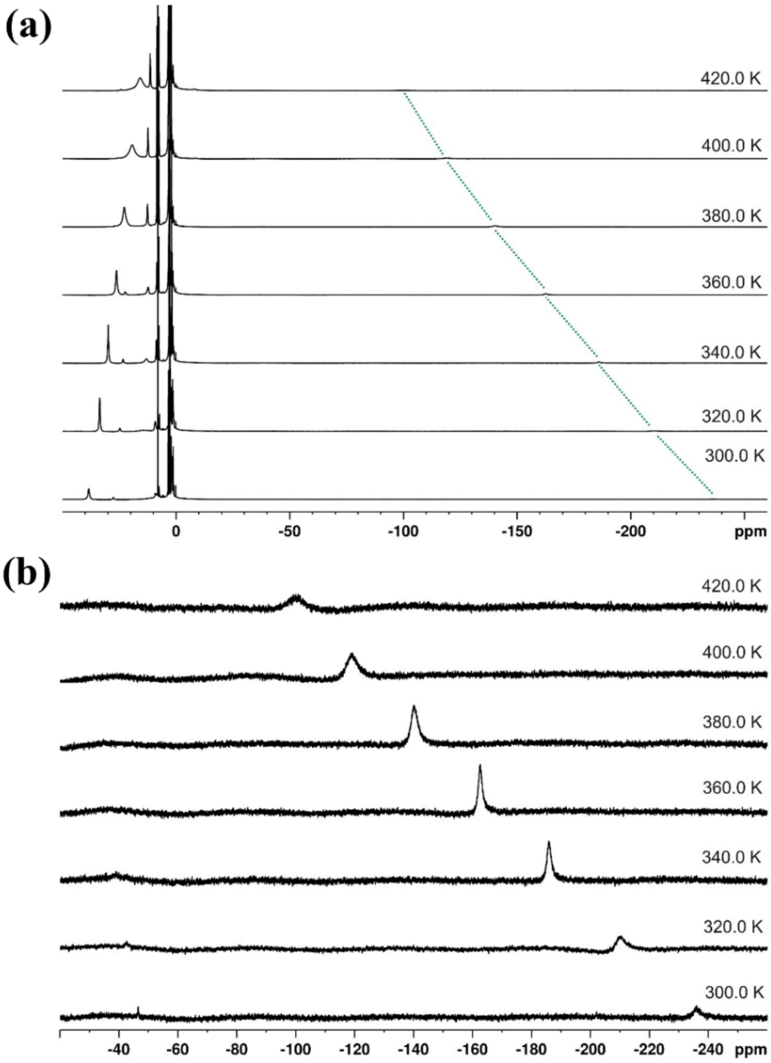

Figure S9. (a) Variable-temperature ^{1}H NMR spectra of **o-1**$\cdot 3CH_3CN\cdot H_2O$ in DMF-d_7. (b) Expanded region (−50 to −260 ppm).

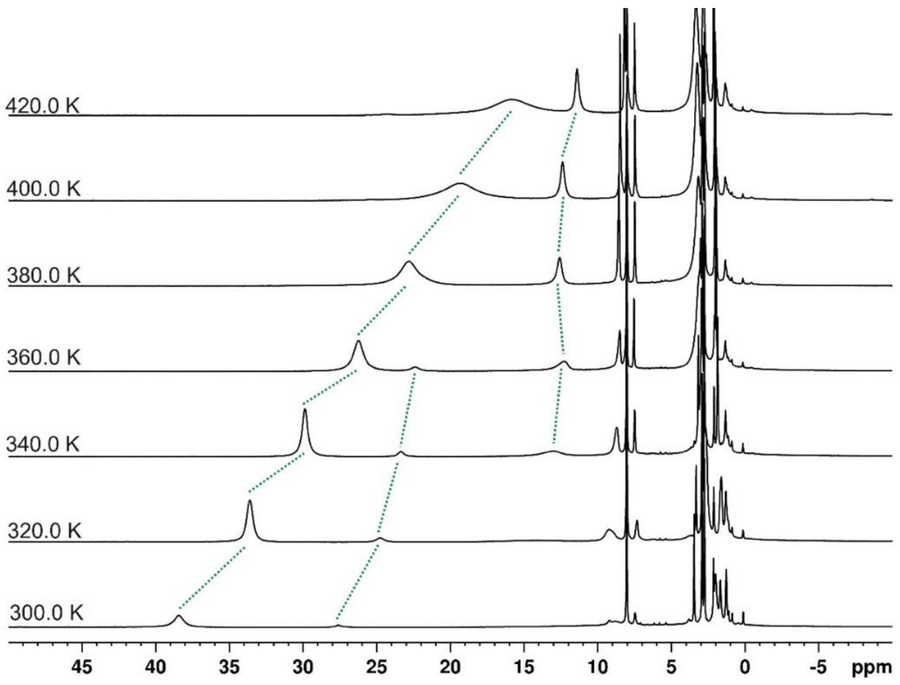

Figure S10. Expanded (+50 to −10 ppm) variable-temperature ^{1}H NMR spectra of **o-1**·$3CH_3CN·H_2O$ in DMF-d_7.

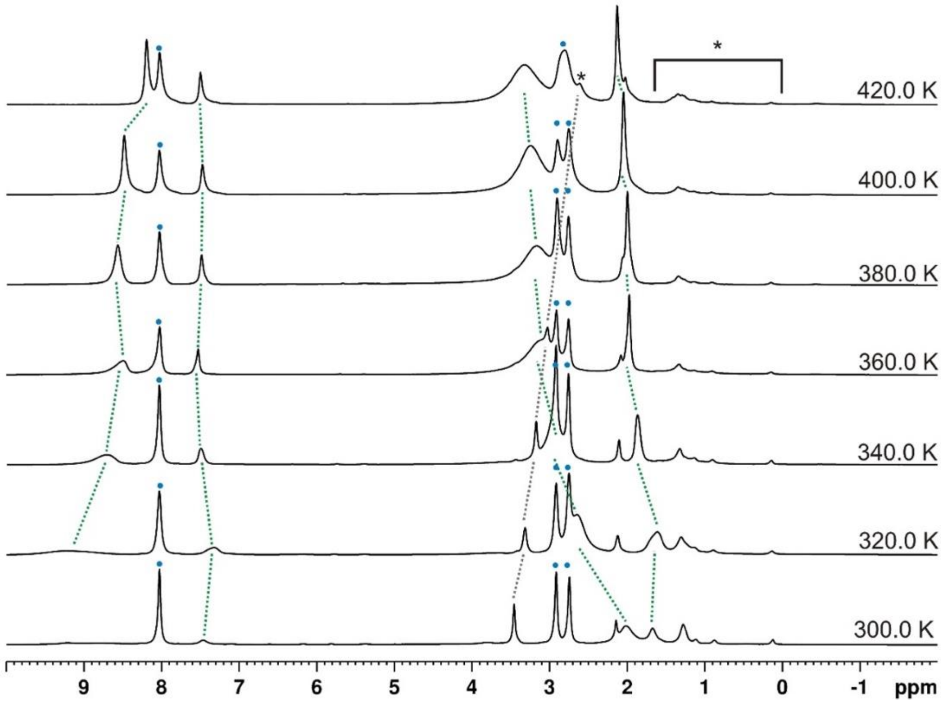

Figure S11. Expanded (+10 to −2 ppm) variable-temperature ^{1}H NMR spectra of **o-1**·$3CH_3CN·H_2O$ in DMF-d_7. Blue dots indicate residual solvent signals. Asterisks (*) indicate impurities.

Studying the photocyclization process by NMR spectroscopy

We next used NMR spectroscopy to examine the photocyclization of the 6F-DAE-py_2 ligand in **o-1**·$3CH_3CN{\cdot}H_2O$ when exposed to ultraviolet light. To this end, we prepared a saturated solution of **o-1**·$3CH_3CN{\cdot}H_2O$ in DMF-d_7 (concentration: approximately 1 mg/mL). The NMR sample was irradiated with UV light for various durations using a handy TLC-visualization lamp (wavelength: 365 nm). Each sample was handled in the absence of visible light to avoid bleaching, and photocyclization was monitored by ^{1}H and ^{19}F NMR spectroscopy (Figure S12 and S13). Photocyclization was followed by ^{19}F NMR spectroscopy, whereas both paramagnetic and diamagnetic regions of the ^{1}H NMR spectrum were used to follow the decomposition of **o-1**·$3CH_3CN{\cdot}H_2O$ over the course of the reaction. The assigned ^{19}F NMR spectrum of the open-form 6F-DAE-py_2 ligand is shown in Figure. S12. Continued irradiation led to a slow increase in the percentage of the closed form of the 6F-DAE-py_2 ligand in **o-1**·$3CH_3CN{\cdot}H_2O$.

For comparison, we acquired ^{19}F NMR spectra of the open and closed forms of 6F-DAE-py_2 (irradiated with UV light for 3 min) and **o-1**·$3CH_3CN{\cdot}H_2O$ (irradiated with UV light for 90 min) in DMF-d_7 at 295 K (Figure S13a). Small differences in chemical shift (on the order of 0.1 ppm) are observable, and the signals of **o-1**·$3CH_3CN{\cdot}H_2O$ are broader owing to paramagnetic relaxation. The photocyclization of **o-1**·$3CH_3CN{\cdot}H_2O$ was 36% complete after 5 min of UV exposure, 82% after 30 min, and more than 85% complete within 45 min of irradiation, based on the intensities of the ^{19}F signals (Figure S13b).

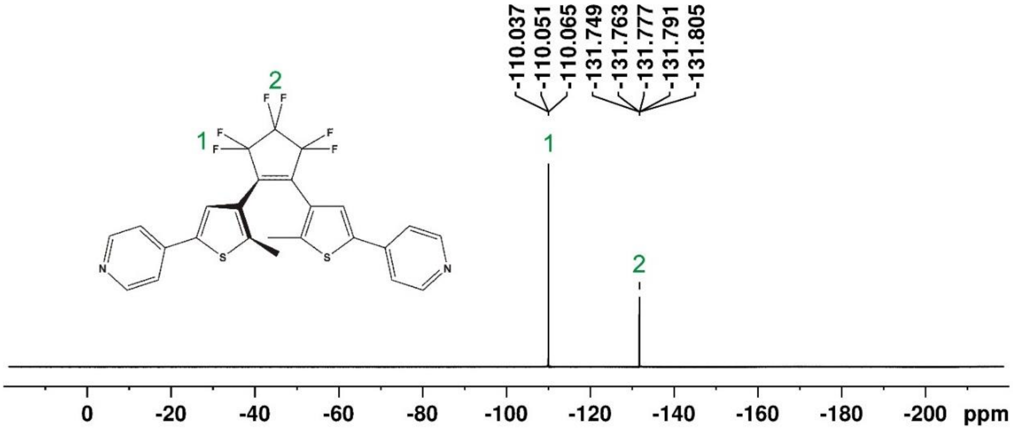

Figure S12. Assigned ^{19}F NMR spectrum of **o-L** in $CDCl_3$ at 295.0 K.

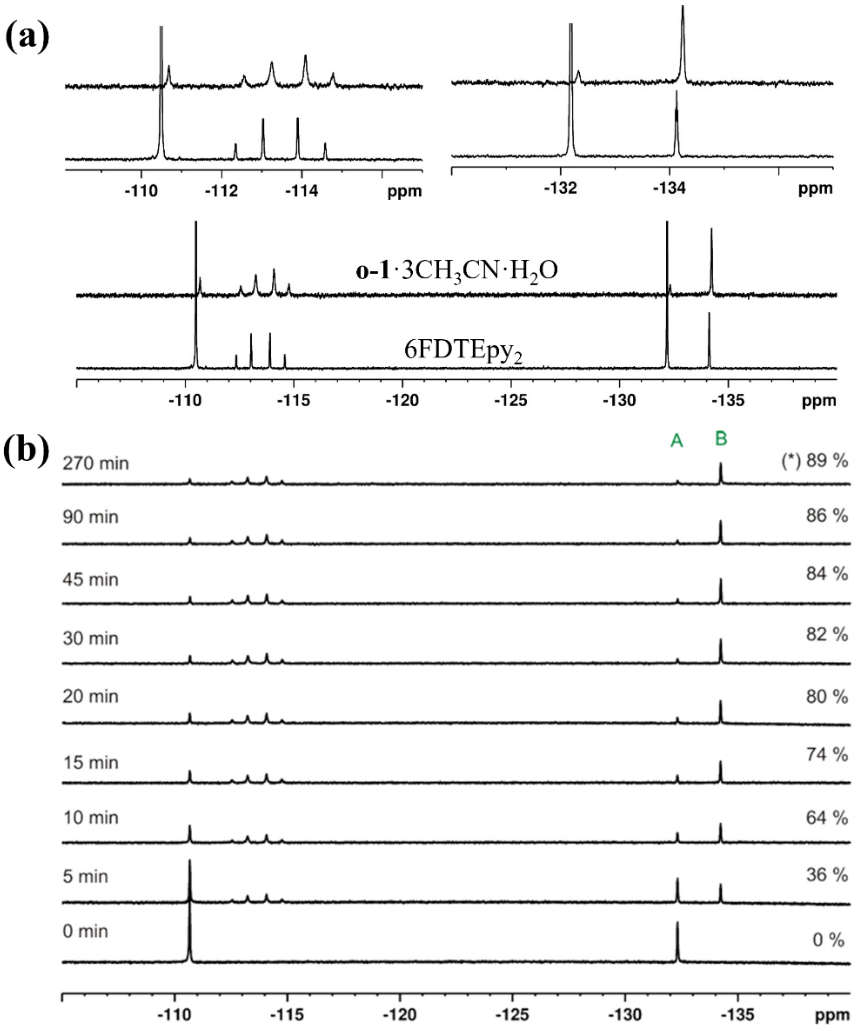

Figure S13. (a) ^{19}F NMR spectra of the open and closed forms of 6F-DAE-py$_2$ (lower spectrum: irradiated with UV light for 3 min) and **o-1**·$3CH_3CN{\cdot}H_2O$ (upper spectrum,

irradiated with UV light for 90 min) in DMF-d_7 at 295 K. The expanded spectra show small differences in the ^{19}F chemical shift (on the order of 0.1 ppm). Upper left: broadened signals of **o-1**·3CH_3CN·H_2O due to paramagnetic relaxation. (b) ^{19}F NMR spectra of **o-1**·3CH_3CN·H_2O acquired following exposure to UV light for different times.

Density functional theory (DFT) calculations for NMR spectroscopy

As shown by Evans, the magnetic moment of the structurally similar [Co(3,5-DTSQ$^{\bullet-}$)(3,5-DTCat^{2-})(4-papy)$_2$] complex (3,5-DTCat^{2-} = 3,5-di-*tert*-butylcatecholate, 3,5-DTSQ$^{\bullet-}$ = 3,5-di-*tert*-butylsemiquinone) increases to 4.00 μ_B at 320 K in toluene solution;(1) this change corresponds to a thermally induced LS-Co^{III}(3,5-DTSQ$^{\bullet-}$)(3,5-DTCat^{2-}) (expected μ_{eff} = 1.7 μ_B) to HS-Co^{II}(3,5-DTSQ$^{\bullet-}$)$_2$ (expected μ_{eff} = 4.34 μ_B) transition. Therefore, we expected **o-1**·3CH_3CN·H_2O to predominantly exist in the HS-Co^{II}(3,5-DTSQ$^{\bullet-}$)$_2$ electronic state at temperatures above 300 K and in benzene solution. A combination of DFT and NMR studies confirmed the expected sextet electronic state of CoL_2 (where L represents the doubly charged 3,5-DTCat^{2-}, singly charged 3,5-DTSQ$^{\bullet-}$, or neutral 3,5-di-tert-butyl-o-benzoquinone chelate ligand) in one-dimensional **o-1**·3CH_3CN·H_2O. Notably, DFT calculations of valence-tautomeric cobalt complexes do not afford reliable energy differences between electronic states; rather, the obtained energy differences should be interpreted qualitatively, as they are known to depend strongly on the choice of the functional used in the calculation.(2) To complement existing calculations with the OPBE functional,(1, 3, 4) we optimized geometries at the B3LYP*/def2-SVP level of theory and calculated energies using the triple-zeta def2-TZVP basis set.(5-9)

Six-coordinate [Co(3,5-DTSQ$^{\bullet-}$)(3,5-DTCat^{2-})(py)$_2$] model complex

DFT geometry optimizations and subsequent broken-symmetry calculations on the [Co(3,5-DTSQ$^{\bullet-}$)(3,5-DTCat^{2-})(py)$_2$] complex led to the following results: A comparison of the optimized HS-Co^{II}(3,5-DTSQ$^{\bullet-}$)$_2$, LS-Co^{II}(3,5-DTSQ$^{\bullet-}$)$_2$, and LS-Co^{III}(3,5-DTSQ$^{\bullet-}$)(3,5-DTCat^{2-}) structures of [Co(3,5-DTSQ$^{\bullet-}$)(3,5-DTCat^{2-})(py)$_2$] revealed that LS-Co^{III}(3,5-DTCat^{2-})(3,5-DTSQ$^{\bullet-}$) is of lowest energy, followed by HS-Co^{II}(3,5-DTSQ$^{\bullet-}$)$_2$, with LS-Co^{II}(3,5-DTSQ$^{\bullet-}$)$_2$ exhibiting the highest energy of the three. Furthermore, among the three optimized systems, LS-Co^{III}(3,5-DTCat^{2-})(3,5-DTSQ$^{\bullet-}$) resembled the crystal structure of **o-1**$\cdot$3$CH_3CN\cdot H_2O$. For comparison, the following average C-O bond lengths were determined: XRD (from the asymmetric unit), 1.884 Å; Co^{III}(3,5-DTCat^{2-})(3,5-DTSQ$^{\bullet-}$), 1.915 Å; HS-Co^{II}(3,5-DTSQ$^{\bullet-}$)$_2$, 2.094 Å; and LS-Co^{II}(3,5-DTSQ$^{\bullet-}$)$_2$, 2.092 Å (averages of 1.979 Å and 2.206 Å).

As mentioned above, we expect the structure equivalent to HS-Co^{II}(3,5-DTSQ$^{\bullet-}$)$_2$ to be present in a solution of **o-1**$\cdot$3$CH_3CN\cdot H_2O$ at 340.0 K. Spin densities obtained from broken-symmetry DFT calculations at the UB3LYP*/def2-TZVP level are shown in Figure S14a. [Co(3,5-DTSQ$^{\bullet-}$)(3,5-DTCat^{2-})(py)$_2$] is slightly higher in energy (~3 kJ/mol) when the dioxolene ligands are cis-arranged rather than trans-arranged. The XRD data show that only the trans isomer was produced. For this reason, and for simplicity, only systems with trans-configured dioxolene ligands were considered when analyzing spin densities. The xyz coordinates of the abovementioned structures are provided along with the calculated energies (Table S4). Positive and negative spin densities are shown in blue and green, respectively, throughout this work.

Three electronic states are possible within the HS-Co^{II}(3,5-DTSQ$^{\bullet-}$)$_2$ structure: a sextet (both π-radicals are ferromagnetically (FM) coupled to high-spin Co^{II}), a quartet (one π-radical is coupled ferromagnetically, the other antiferromagnetically (AFM) to high-spin Co^{II}), and a doublet (both π-radicals are antiferromagnetically coupled to high-spin Co^{II}). Broken-

symmetry DFT calculations show that the sextet state has the lowest energy. Spin-density diagrams of the three electronic states are shown in Figure S14b.

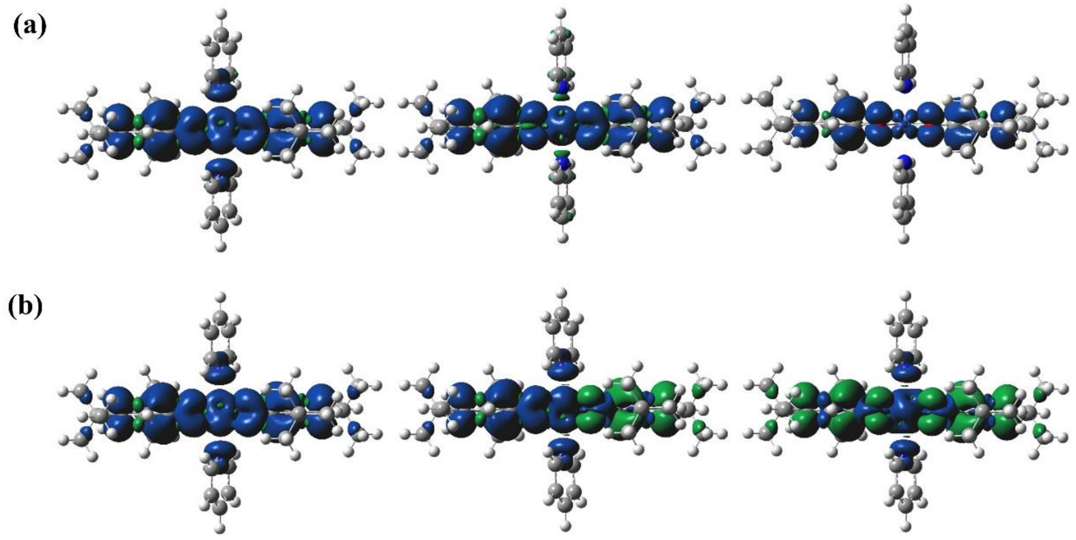

Figure S14. (a) UB3LYP*/def2-TZVP-calculated spin-density maps (isovalue 0.0008 a.u.) of the [Co(3,5-DTSQ$^{\bullet-}$)(3,5-DTCat^{2-})(py)$_2$] complex (trans-isomer) in the HS-Co^{II}(3,5-DTSQ$^{\bullet-}$)$_2$, LS-Co^{II}(3,5-DTSQ$^{\bullet-}$)$_2$, and LS-Co^{III}(3,5-DTCat^{2-})(3,5-DTSQ$^{\bullet-}$) structures (left to right, respectively, with trans-configured dioxolene ligands). The dioxolene ligands are in the horizontal plane. (b) Spin-density maps (isovalue 0.0008 a.u.) of the [Co(3,5-DTSQ$^{\bullet-}$)(3,5-DTCat^{2-})(py)$_2$] complex (trans-isomer, HS-Co^{II}(3,5-DTSQ$^{\bullet-}$)$_2$ structure) in sextet, quartet, and doublet electronic states (left to right, respectively, with trans-configured dioxolene ligands).

Table S4. Calculated energies for various electronic states of high-spin and low-spin [Co(3,5-DTSQ$^{\bullet-}$)(3,5-DTCat^{2-})(py)$_2$] with trans-configured dioxolene ligands

**[Co(3,5-DTSQ$^{\bullet-}$)(3,5-DTCat^{2-})(py)$_2$] (trans-isomer, HS-Co^{II}(3,5-DTSQ$^{\bullet-}$)$_2$)**	
Energy (sextet)	−3256.28747192 E_h (UB3LYP*/def2-TZVP)
Energy (quartet)	− 3256.28537207 E_h

Energy (doublet)	−3256.28183813 E_h
**[Co(3,5-DTSQ$^{\bullet-}$)(3,5-DTCat^{2-})(py)$_2$] (trans-isomer, LS-Co^{II}(3,5-DTSQ$^{\bullet-}$)$_2$)**	
Energy (quartet)	−3256.28024246 E_h (UB3LYP*/def2-TZVP)
**[Co(3,5-DTSQ$^{\bullet-}$)(3,5-DTCat^{2-})(py)$_2$] (trans-isomer, LS-Co^{III}(3,5-DTCat^{2-})(3,5-DTSQ$^{\bullet-}$))**	
Energy (doublet)	−3256.29175494 E_h (UB3LYP*/def2-TZVP)

Five-coordinate [Co(3,5-DTSQ$^{\bullet-}$)(3,5-DTCat^{2-})(py)] model complex

The geometry of [Co(3,5-DTSQ$^{\bullet-}$)(3,5-DTCat^{2-})(py)] was optimized in the HS-Co^{II}(3,5-DTSQ$^{\bullet-}$)$_2$ sextet state. Broken-symmetry calculations revealed that this electronic state has the same energy (to within error) as the doublet electronic state, in which π-radicals couple antiferromagnetically to high-spin Co^{II}. The quartet electronic state, in which the two ligand radicals are antiferromagnetically coupled, is somewhat lower in energy than the other two electronic states. The cis-dioxolene ligand configuration leads to a structure with a slightly higher energy than that of the trans configuration but similar to the six-coordinate [Co(3,5-DTSQ$^{\bullet-}$)(3,5-DTCat^{2-})(py)$_2$]. The xyz coordinates of the optimized [Co(3,5-DTSQ$^{\bullet-}$)(3,5-DTCat^{2-})(py)] structures and the calculated energies are provided here (Table S5). Spin-density maps of the sextet, quartet, and doublet electronic states are shown in Figure. S15. Existing experimental data for a [Co(3,5-DTSQ$^{\bullet-}$)(3,5-DTCat^{2-})L] complex, in which L is a monodentate imine ligand, show that these complexes are bis-semiquinonato high-spin Co^{II} complexes, with a total *S* of 3/2 due to antiferromagnetically coupled radical ligands.(10) Antiferromagnetic coupling between two ligand radicals and a high-spin Co^{II} state has also been observed in a distorted trigonal-prismatic complex.(11)

Based on existing experimental and theoretical data, we conclude that a *β*-spin on one of the semiquinonato ligands leads to Fermi contact shifts of opposite sign to those observed for the six-coordinate compound (in the one-dimensional chain) if a significant amount of the five-coordinate decomposition product is present in a solution of **o-1**·$3CH_3CN{\cdot}H_2O$. Hence, the presence of the five-coordinate species is easily identified by the negatively shifted tBu-group signal or the positively shifted *meta*-^{1}H signal of the semiquinonato ligand. Consequently, this information can be used to qualitatively confirm the main species present in an **o-1**·$3CH_3CN{\cdot}H_2O$ solution.

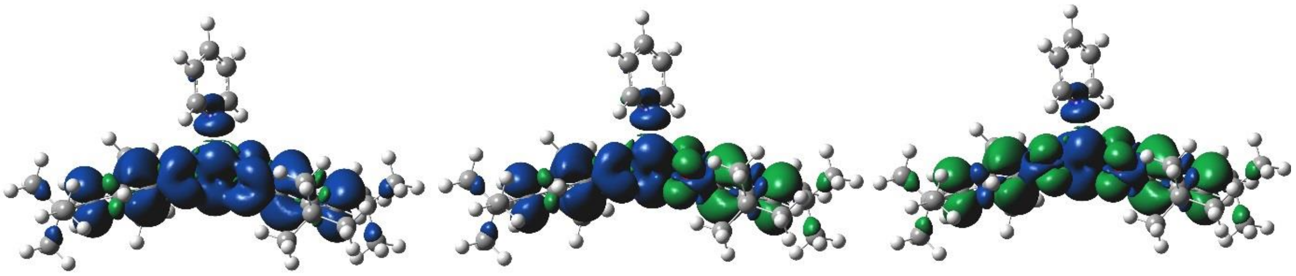

Figure S15. UB3LYP*/def2-TZVP-calculated spin-density maps (isovalue 0.0008 a.u.) of the [Co(3,5-DTSQ$^{\bullet-}$)(3,5-DTCat^{2-})(py)] complex (trans-isomer) in the HS-Co^{II}(3,5-DTSQ$^{\bullet-}$)$_2$ structure in its sextet, quartet, and doublet electronic states (left to right, respectively).

Table S5. Calculated energies of trans-configured high-spin and low-spin [Co(3,5-DTSQ$^{\bullet-}$)(3,5-DTCat^{2-})(py)] in various electronic states.

**[Co(3,5-DTSQ$^{\bullet-}$)(3,5-DTCat^{2-})(py)] (trans-isomer, HS-Co^{II}(3,5-DTSQ$^{\bullet-}$)$_2$)**	
Energy (sextet)	−3009.60395725 E_h (UB3LYP*/def2-TZVP)
Energy (quartet)	−3009.60496260 E_h
Energy (doublet)	−3009.60300181 E_h

[Co(3,5-DTSQ$^{\bullet-}$)(3,5-DTCat^{2-})(py)] (cis-isomer, HS-Co^{II}(3,5-DTSQ$^{\bullet-}$)$_2$)	
Energy (doublet)	−3009.60212532 E_h (UB3LYP*/def2-TZVP)

Six-coordinate [Co(3,5-DTSQ$^{\bullet-}$)(3,5-DTCat^{2-})(6F-DAE-py$_2$)$_2$] model complex

Based on the model five- and six-coordinate complex calculations, the trans isomers of the [Co(3,5-DTSQ$^{\bullet-}$)(3,5-DTCat^{2-})(6F-DAE-py$_2$)$_2$] and [Co(3,5-DTSQ$^{\bullet-}$)(3,5-DTCat^{2-})(6F-DAE-py$_2$)] structures were optimized in their sextet electronic states (as HS-Co^{II}(3,5-DTSQ$^{\bullet-}$)$_2$), and the optimized structures were used to calculate the spin densities at the UB3LYP*/def2tzvp level (sextet electronic state for [Co(3,5-DTSQ$^{\bullet-}$)(3,5-DTCat^{2-})(6F-DAE-py$_2$)$_2$] and quartet electronic state for [Co(3,5-DTSQ$^{\bullet-}$)(3,5-DTCat^{2-})(6F-DAE-py$_2$)]. A comparison of the calculated spin densities of the five- and six-coordinate structures enabled the main species responsible for the ^{1}H NMR spectrum of **o-1**·3CH_3CN·H_2O in DMF-d_7 to be unambiguously identified. Spin-density maps of the [Co(3,5-DTSQ$^{\bullet-}$)(3,5-DTCat^{2-})(6F-DAE-py$_2$)$_2$] and [Co(3,5-DTSQ$^{\bullet-}$)(3,5-DTCat^{2-})(6F-DAE-py$_2$)] structures are shown in Figure S16, and the xyz coordinates of these structures are provided in Table S6, along with the calculated energies.

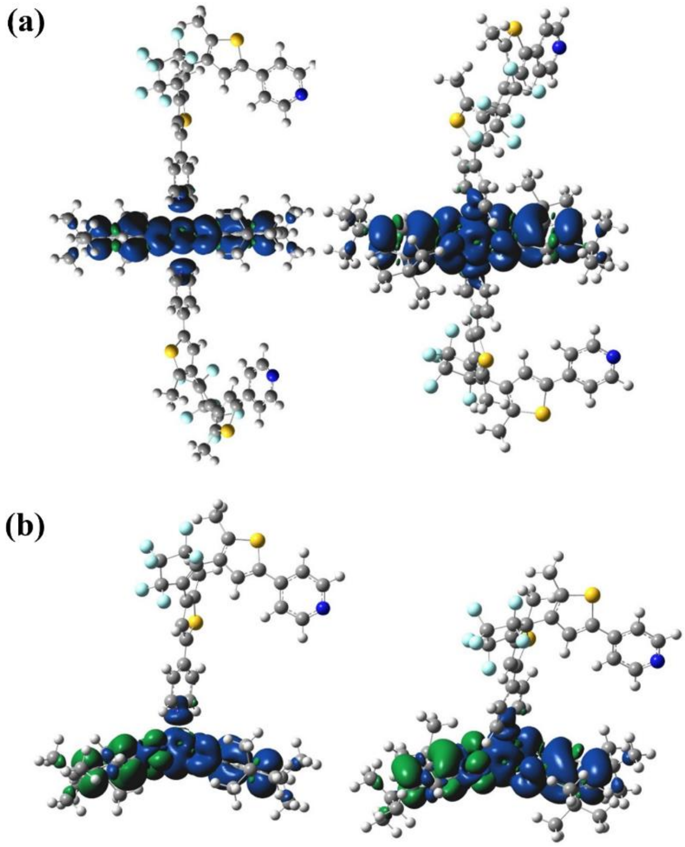

Figure S16. (a) UB3LYP*/def2-TZVP-calculated spin-density maps of (a) [Co(3,5-DTSQ$^{\bullet-}$)(3,5-DTCat^{2-})(6F-DAE-py$_2$)$_2$] (trans-isomer, open form, sextet; isovalue 0.0008 a.u.) and (b) [Co(3,5-DTSQ$^{\bullet-}$)(3,5-DTCat^{2-})(6F-DAE-py$_2$)] (trans isomer, quartet; isovalue 0.0008 a.u.). Left: side views; right: tilted side views.

Table S6. Calculated energies of trans-configured high-spin and low-spin [Co(3,5-DTSQ$^{\bullet-}$)(3,5-DTCat^{2-})(6F-DAE-py$_2$)$_2$] in various electronic states

**[Co(3,5-DTSQ$^{\bullet-}$)(3,5-DTCat^{2-})(6F-DAE-py$_2$)$_2$] (trans-isomer, 6F-DAE-py$_2$ “open”, HS-Co^{II}(3,5-DTSQ$^{\bullet-}$)$_2$)**	
Energy (sextet)	−7672.94458012 E_h (UB3LYP*/def2-TZVP)
**[Co(3,5-DTSQ$^{\bullet-}$)(3,5-DTCat^{2-})(6F-DAE-py$_2$)] (trans-isomer, 6F-DAE-py$_2$ “open”, HS-**	

Co^{II}(3,5-DTSQ$^{•-}$)$_2$)	
Energy (quartet)	− 5217.93342556 E_h (UB3LYP*/def2-TZVP)

^{1}H spin densities were calculated based on the optimized structures and electronic states (average values are provided for the methyl and tBu groups). Table S7 reveals that the β-spin semiquinone ligand exhibits spin densities of approximately the same magnitude as those of the α-spin semiquinone ligand but with opposite signs. Consequently, the presence of the five-coordinate dissociation product of **o-1**·3CH_3CN·H_2O in solution can easily be identified by NMR spectroscopy (i.e., one sharp signal at approximately +250 ppm (m-aSQ) and one intense broad signal at approximately −30 ppm due to the m-aSQ-tBu group; chemical shifts were estimated from the experimental hyperfine ^{1}H NMR shifts of the corresponding groups at 343 K). Naming schemes for the six- and five-coordinate complexes are shown in Figure S17.

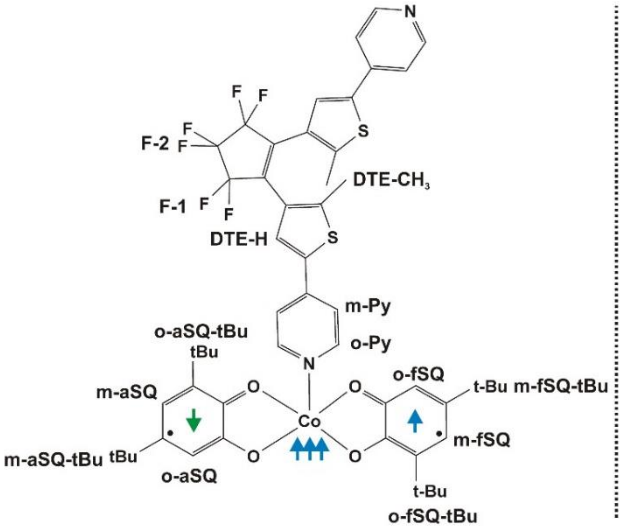

Figure S17. Naming scheme for the various ^{1}H and ^{19}F resonances in the spectra of [Co(3,5-DTSQ$^{\bullet-}$)(3,5-DTCat^{2-})(6F-DAE-py$_2$)] (left, five-coordinate) and [Co(3,5-DTSQ$^{\bullet-}$)(3,5-DTCat^{2-})(6F-DAE-py$_2$)$_2$] (right, six-coordinate).

Table S7. Calculated ^{1}H and ^{19}F nuclear spin densities (a.u.) in [Co(3,5-DTSQ$^{\bullet-}$)(3,5-DTCat^{2-})(6F-DAE-py$_2$)$_2$] (trans-isomer, sextet) and [Co(3,5-DTSQ$^{\bullet-}$)(3,5-DTCat^{2-})(6F-DAE-py$_2$)] (trans-isomer, quartet)

**[Co(3,5-DTSQ$^{\bullet-}$)(3,5-DTCat^{2-})(6F-DAE-py$_2$)]**		**[Co(3,5-DTSQ$^{\bullet-}$)(3,5-DTCat^{2-})(6F-DAE-py$_2$)$_2$]**	
Group	Spin Density $\times 10^5$ [a.u.]	Group	Spin Density $\times 10^5$ [a.u.]
6F-DAE-CH$_3$	−4.0	6F-DAE-CH$_3$	−2.3
6F-DAE-H	3.0	6F-DAE-H	3.0
m-Py	19.0	*m*-Py	22.0
o-Py	69.0	*o*-Py	60.0
o-fSQ	2.0	*o*-SQ	−2.0
o-fSQ-tBu	3.2	*o*-SQ-tBu	3.2
m-fSQ	−132.0	*m*-SQ	−127.5
m-fSQ-tBu	22.8	*m*-SQ-tBu	15.9
o-aSQ	19.0	F-1	0.0
o-aSQ-tBu	−2.3	F-2	0.0
m-aSQ	179.0		
m-aSQ-tBu	−23.8		
F-1	0.0		
F-2	0.0		

The high-resolution ^{1}H NMR spectrum of **o-1**·$3CH_3CN·H_2O$ in DMF-d_7 shown in Figure S18 reveals very weak signals at −13.8 and −38.9 ppm. No signal was observed at approximately +250 ppm. We assume that these signals correspond to terminal protons in the one-dimensional **o-1**·$3CH_3CN·H_2O$ chains (m-aSQ-tBu and o-aSQ-tBu), which are possibly five-coordinate HS-Co^{II} centers because the terminal 6F-DAE-py_2 ligands can dissociate. These signals enable the average length of a single one-dimensional **o-1**·$3CH_3CN·H_2O$ chain to be estimated; integration and comparison with the m-SQ-tBu signal provided a ratio of approximately 1:190; hence, we estimate that there are approximately 190 Co(II) centers within a single **o-1**·$3CH_3CN·H_2O$ chain.

It should be noted that the spin densities calculated for the individual ^{1}H nuclei of the tBu groups in the optimized complexes range in value and are averaged in Table S7. Furthermore, the five- and six-coordinate structures have three unpaired electron sources (two ligand radicals and one metal ion). Therefore, the tabulated spin density data should be interpreted semi-quantitatively, with the usual equations for Fermi contact shifts not used in these analyses.(12) Nevertheless, the m-SQ-tBu- and m-SQ-proton signals serve as the most reliable identifiers of **o-1**·$3CH_3CN·H_2O$ in solution.

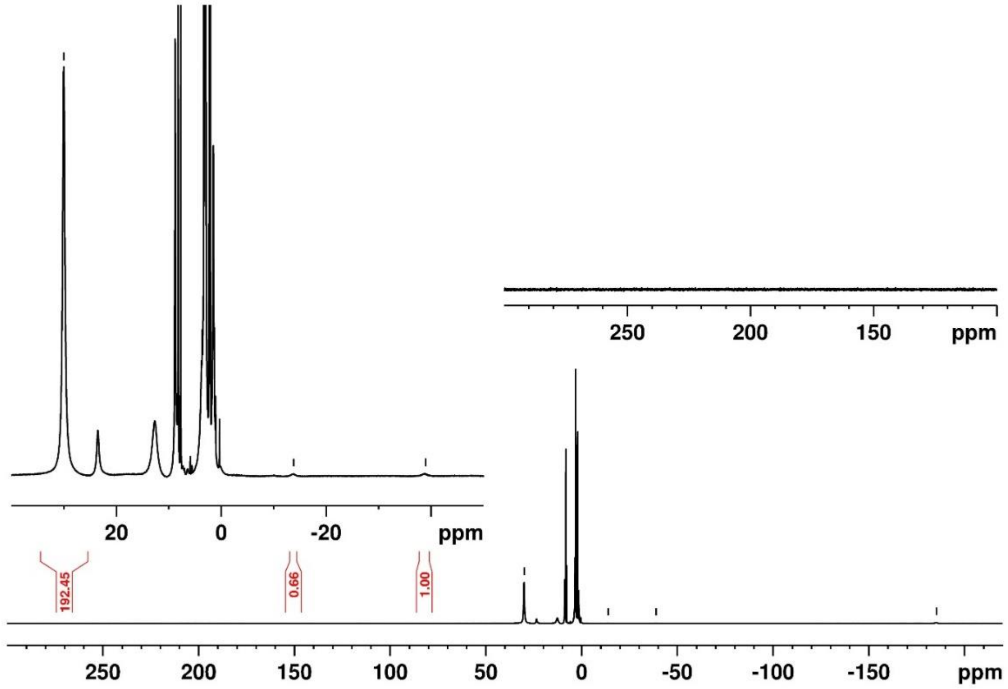

Figure S18. High-resolution ^{1}H NMR spectrum of **o-1**$\cdot 3CH_3CN\cdot H_2O$ in DMF-d_7 recorded at 343 K in a 14.09 T field. Expanded regions: between +40 and −50 ppm (left) and +300 and +100 ppm (right). Selected signal intensities are shown below the left expanded spectrum.

Further discussion from NMR measurements and DFT calculations

The ^{1}H NMR spectrum of **o-1**$\cdot 3CH_3CN\cdot H_2O$ and the calculated spin densities of the [Co(3,5-DTSQ$^{\bullet-}$)(3,5-DTCat^{2-})(6F-DAE-py$_2$)$_2$] complex used for signal assignment show that the resonances associated with the pyridine moieties of the 6F-DAE-py$_2$ ligand have smaller hyperfine shifts compared to those calculated from the spin densities. **o-1**$\cdot 3CH_3CN\cdot H_2O$ is a valence-tautomeric compound and is expected to transition from the LS-Co^{III}(3,5-DTCat^{2-})(3,5-DTSQ$^{\bullet-}$) to the HS-Co^{II}(3,5-DTSQ$^{\bullet-}$)$_2$ state in solution at approximately room temperature.(1) Some of the paramagnetic signals of **o-1**$\cdot 3CH_3CN\cdot H_2O$ exhibited non-Curie behavior when the temperature was varied (see below), consistent with this compound undergoing the abovementioned transition. The calculated spin densities associated with the

^{1}H resonances of the pyridine ligands in the [Co(3,5-DTSQ$^{\bullet-}$)(3,5-DTCat^{2-})(py)$_2$] model complexes are compared in Table S8; the listed values were calculated at the UB3LYP*/def2-TZVP level of theory. The data reveal considerable differences between the spin densities of the HS-Co^{II}(3,5-DTSQ$^{\bullet-}$)$_2$ and LS-Co^{III}(3,5-DTCat^{2-})(3,5-DTSQ$^{\bullet-}$) states in the six-coordinate model complex. The transition from the LS-Co^{III}(3,5-DTCat^{2-})(3,5-DTSQ$^{\bullet-}$) state to the HS-Co^{II}(3,5-DTSQ$^{\bullet-}$)$_2$ state led to these signals showing non-Curie behavior and lower-than-expected chemical shifts compared to those of the pure HS-Co^{II}(3,5-DTSQ$^{\bullet-}$)$_2$ state.

Table S8. Spin densities (a.u.) of the HS-Co^{II}(3,5-DTSQ$^{\bullet-}$)$_2$, LS-Co^{II}(3,5-DTSQ$^{\bullet-}$)$_2$, and LS-Co^{III}(3,5-DTCat^{2-})(3,5-DTSQ$^{\bullet-}$) states of the model [Co(3,5-DTSQ$^{\bullet-}$)(3,5-DTCat^{2-})(py)$_2$] complex

	HS-Co^{II}(3,5-DTSQ$^{\bullet-}$)$_2$	LS-Co^{II}(3,5-DTSQ$^{\bullet-}$)$_2$	LS-Co^{III}(3,5-DTCat^{2-})(3,5-DTSQ$^{\bullet-}$)
Group	Spin Density $\times 10^5$ [a. u.]		
o-Py	61	14	1
m-Py	25	–2	–1

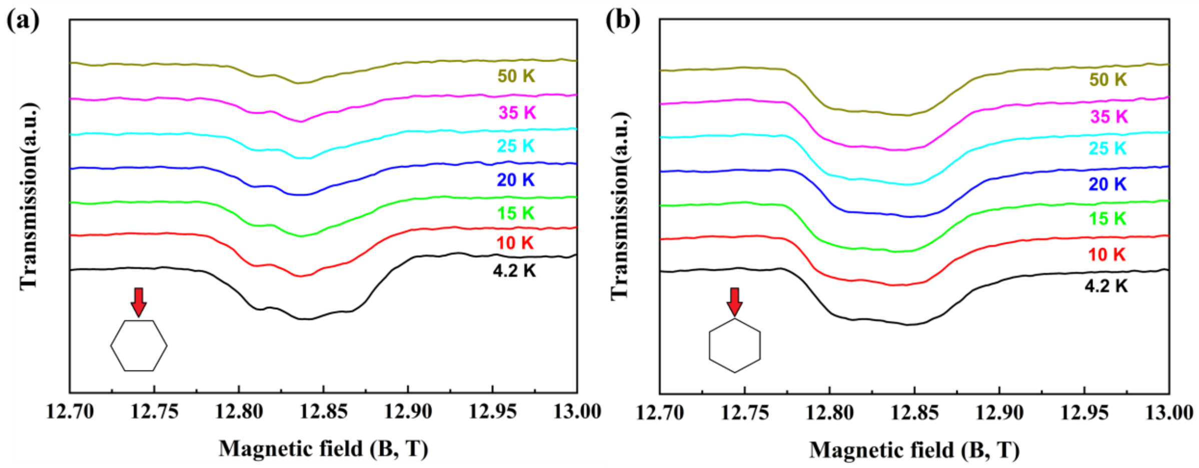

Figure S19. High-field (~13.3 T) and high-frequency (~360 GHz) single-crystal EPR spectra of **o-1**·$3CH_3CN{\cdot}H_2O$ when a static field is directed from the (a) side and (b) corner of the *ab* plane.

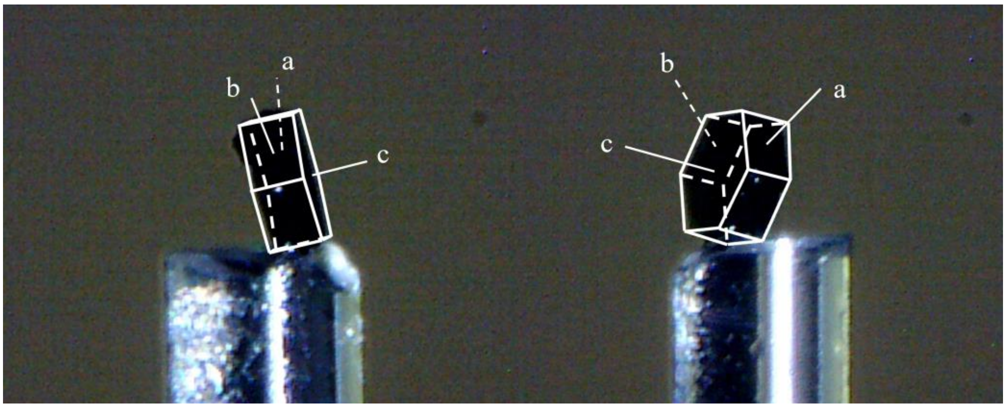

Figure S20. Other face indexes of a single crystal of **o-1**·$3CH_3CN{\cdot}H_2O$.

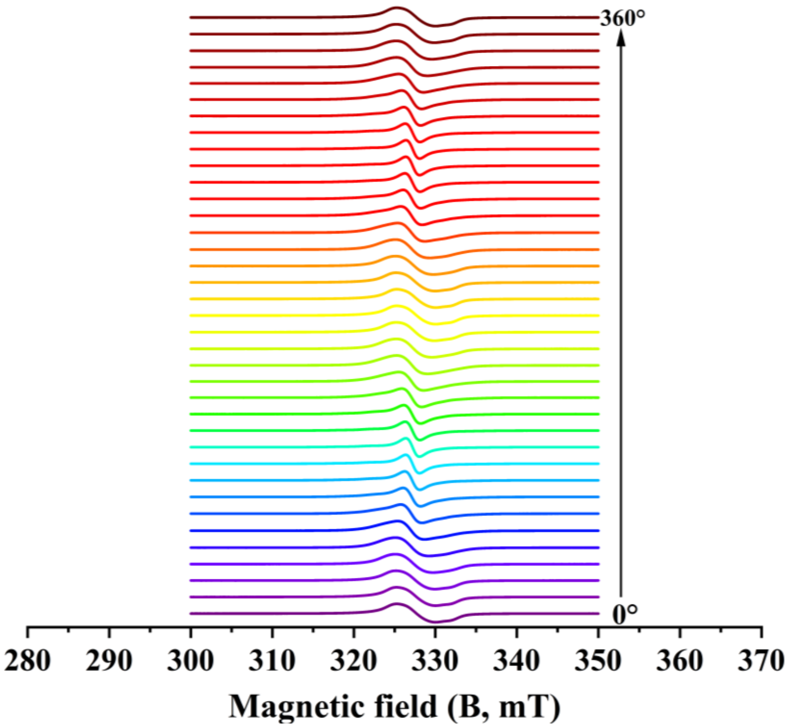

Figure S21. Angular dependence of the X-band EPR spectrum acquired at 100 K along the *c* axis of a single crystal of **o-1**·$3CH_3CN{\cdot}H_2O$ (defined as 0 °).

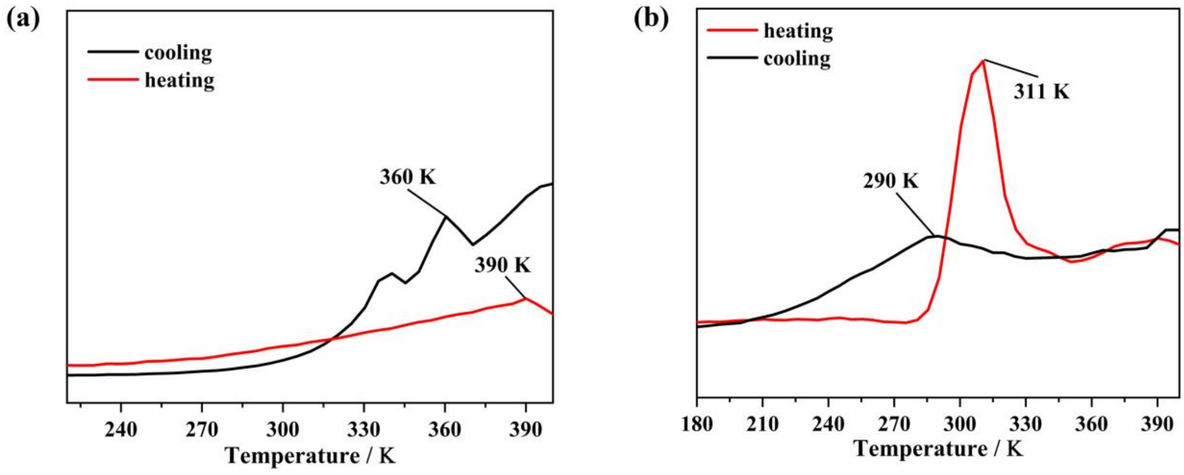

Figure S22. First-order derivatives of $\chi_m T$ for (a) **o-1**·$3CH_3CN\cdot H_2O$ and (b) **c-1**·H_2O.

Thermogravimetric analysis shows the loss of solvent molecules and degradation. **o-1**·$3CH_3CN\cdot H_2O$ started losing weight as soon as it was heated and lost 7.69% weight after heating to 487 K. The first period below 353 K can be attributed to the loss of free solvent molecules on the crystal surface. The following weight loss was due to the loss and partial loss of water and acetonitrile molecules of the complex (the loss of one water molecule and one acetonitrile molecule corresponds to 5.1%). In addition, **c-1**·H_2O lost 0.91% weight when the temperature reached 553 K because of the absence of crystalline solvent molecules.

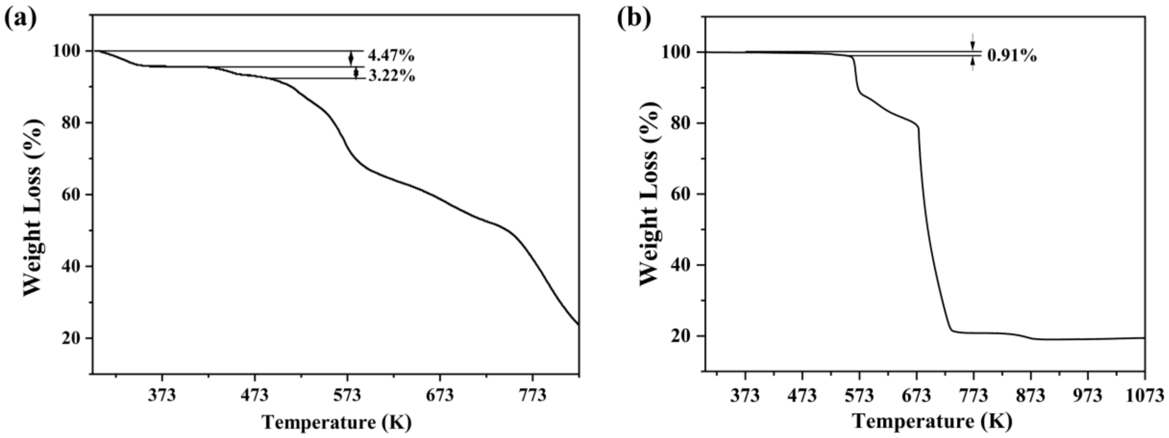

Figure S23. Thermogravimetric analysis of (a) **o-1**·$3CH_3CN\cdot H_2O$ and (b) **c-1**·H_2O.

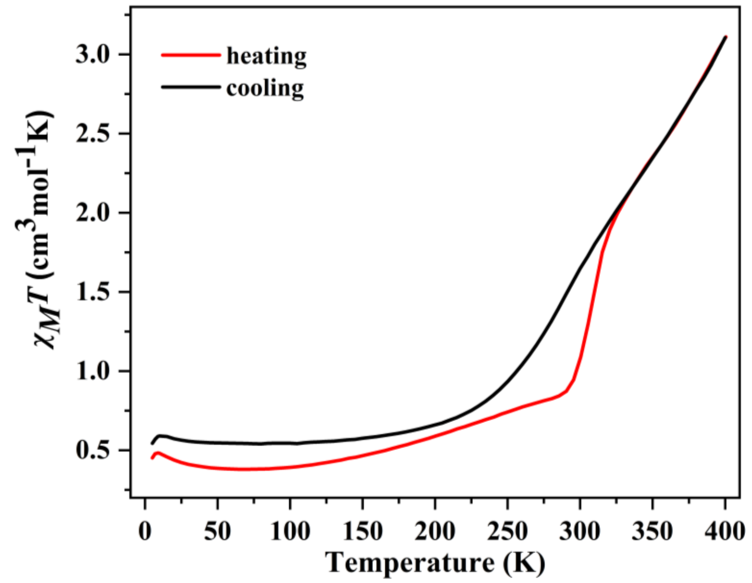

Figure S24. Magnetic susceptibility of **c-1** H_2O. The black curve corresponds to the heating process of a fresh sample containing crystal solvents; the red curve corresponds to the cooling process after solvent loss.

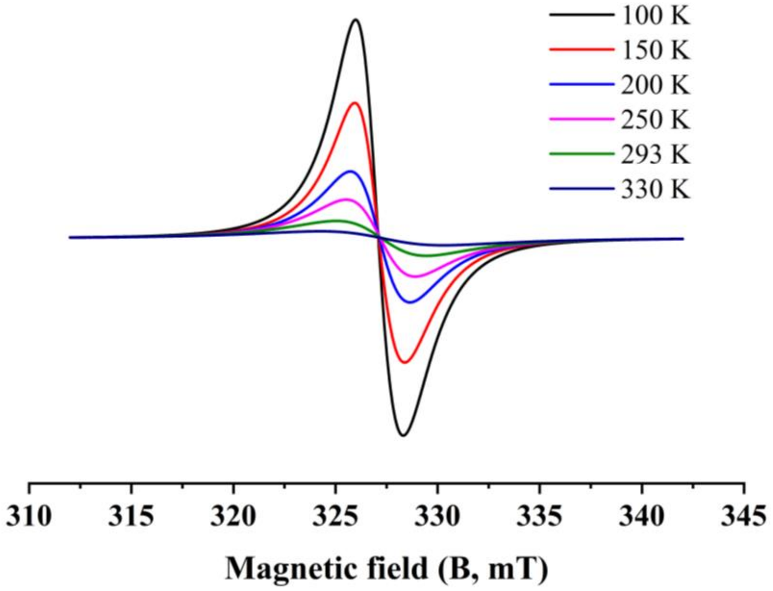

Figure S25. X-band (9.1 GHz) EPR spectra of **c-1** H_2O in the polycrystalline state acquired at various temperatures, which shows the very typical line shape of the SQ˙ radical but a lack of hyperfine interactions.

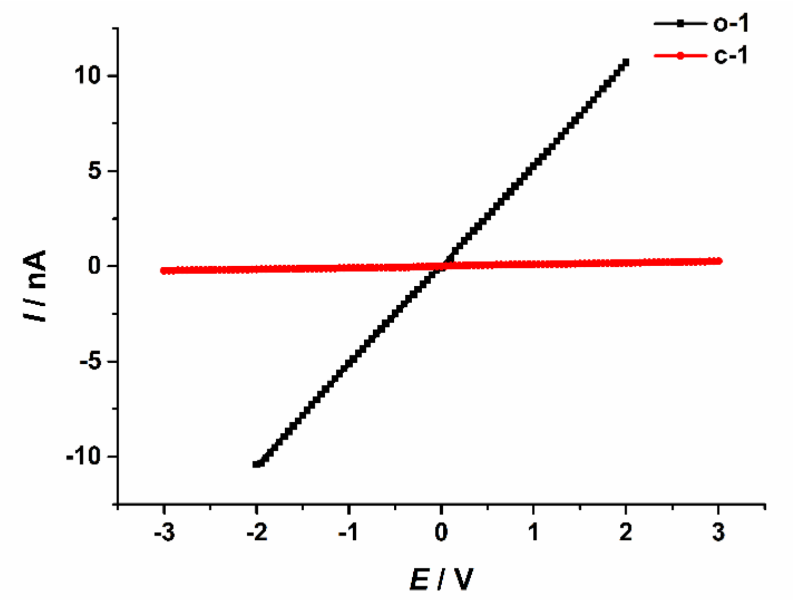

Figure S26. Electrical conductivity (I–V) curves for **o-1**·3CH_3CN·H_2O and **c-1** ·H_2O at 300 K reveal that **c-1** ·H_2O is an extremely poorly conductive insulator.

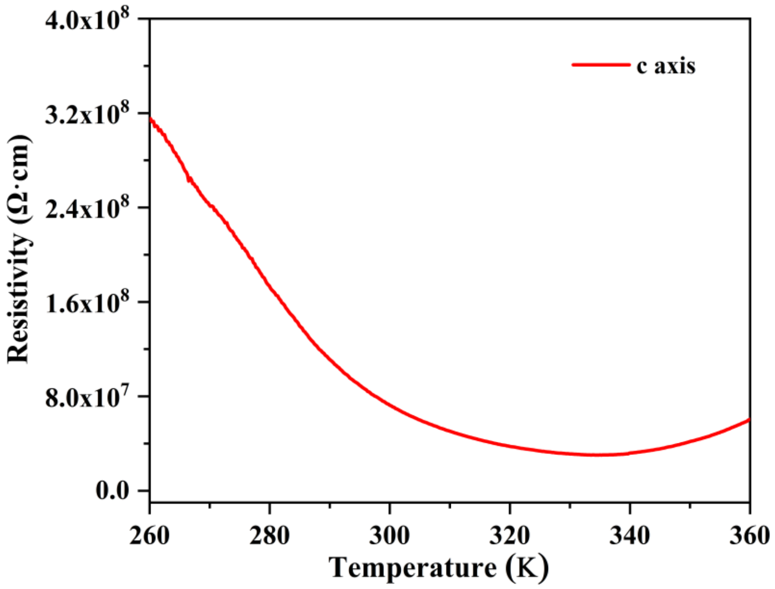

Figure S27. Resistivity of **o-1**·3CH_3CN·H_2O along the *c*-axis as a function of temperature (260–360 K).

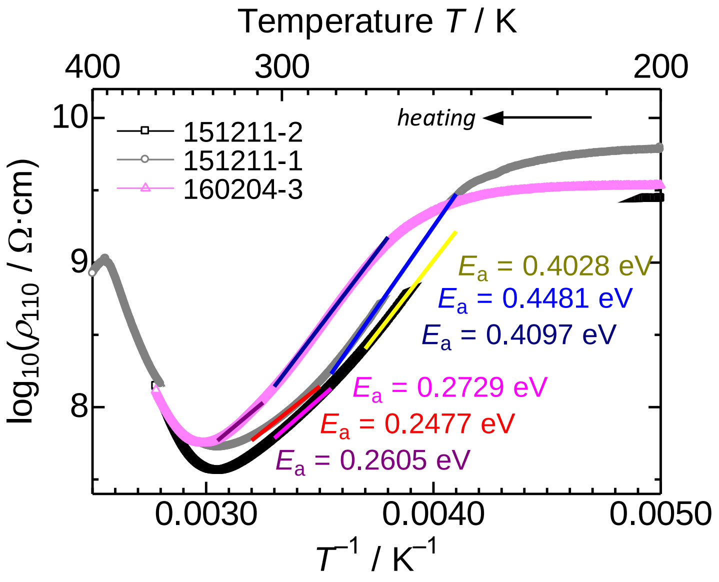

Figure S28. Resistivity vs. 1/T curves for the three investigated samples.

Table S9. Activation energies for three samples in different temperature regions and crossover temperatures

**Sample**	$\boldsymbol{E_a}$ **(LT)/eV**	$\boldsymbol{E_a}$ **(HT)/eV**	**Crossover *T*/K**
1	0.4481<br>(246–270 K)	0.2477<br>(283–302 K)	329
2	0.4028<br>(248–276 K)	0.2729<br>(289–300 K)	329
3	0.4097<br>(270–297 K)	0.2605<br>(308–321 K)	335

Density functional theory (DFT) treatment of the conductivity section

Method: System models were built from the X-ray-diffractometry-determined structures harvested for the closed (100 K, 380 K) and open (100 K, 400 K) forms. Disorder information was manually removed by retaining the selected conformational isomer of a certain group. Calculations on the isolated systems were performed with Gaussian 16,(13) using DFT and HF methods and the def2-TZVP basis set, unless otherwise mentioned. A superfine integration grid was applied for numerical integration, and a tight convergence threshold (10^{-8} for the root mean square change in the density matrix) was used in the self-consistent field (SCF) procedure. DFT calculations with periodic boundary conditions (PBCs) were performed using the VASP 5.4.4 package with the Perdew–Burke–Ernzerhof (PBE) exchange–correlation functional.(14-18) Projector augmented wave (PAW) pseudopotentials and plane-wave basis sets with cutoff energies of 640 eV were used.(19) A Hubbard effective parameter (*U*) of 6.0 eV was applied to the *d* electrons of cobalt;(20) this value was determined as described below (*vide infra*). A $2 \times 2 \times 2$ gamma-centered *k*-point grid was used in the self-consistent parts of the band-structure calculations for the open form **o-1**·$3CH_3CN$·H_2O, with Gaussian smearing (σ = 0.02 eV). The spin configuration was initialized according to the electronic configuration of LS-Co^{III}(3,5-DTCat^{2-})(3,5-DTSQ$^{\bullet-}$), which is the low-spin ground state of the CoL_2 unit, at low temperatures. Molecules and isosurfaces were visualized using Multiwfn 3.8 and VMD 1.9.3.(21, 22) Isosurface values were set to 0.02 a.u. for molecular orbitals and 0.005 a.u. for spin densities, unless otherwise specified.

We first explore the electronic structure of the neutral tautomeric building block (CoL_2). Unlike conventional spin crossover complexes, the CoL_2 building block exhibits valence-tautomeric interconversion, in which the transition between two spin states cooperates with

the metal-to-ligand charge transfer (MLCT) process. Consequently, the oxidation number of the metal center is different after the transition: it is in the Co^{III}(3,5-DTCat^{2-})(3,5-DTSQ$^{\bullet-}$) low-spin state at low temperature and the Co^{II}(3,5-DTSQ$^{\bullet-}$)(3,5-DTSQ$^{\bullet-}$) high-spin state at high temperatures. Therefore, the method selected (exchange–correlation functional) for calculating the electronic structure needs to describe both cases fairly and be able to correctly distinguish the preferred low-spin and high-spin electronic configurations in the corresponding low- and high-temperature molecular structures. The multiple oxidation numbers and spin multiplicities of the fragments, combined with different magnetic coupling scenarios, result in eight possible electronic configurations, as listed in Table S10.

Single-point Kohn–Sham-DFT (KS-DFT) energies were calculated using various functionals for both the low- (100 K) and high-temperature (380 K) structures of the CoL_2 (Co_2) unit from **o-1**·$3CH_3CN$·H_2O. The listed configurations were used as initial guesses for symmetry-breaking DFT calculations. The results show that the exact–exchange admixture is able to stabilize the high-spin state (VII) as well as the metastable state (IV); however, the energy of the metastable state should be slightly higher than those of the low-spin and high-spin states; consequently, B3LYP with a 20% exact–exchange admixture is suitable for the CoL_2 system (Table S11).

Table S10. Plausible initial guesses for the electronic configuration of the neutral CoL_2 unit

Guess	Co		L1	L2	Configuration
I(ls)	III	LS	0	FM	LS-Co^{III}(3,5-DTCat^{2-})(3,5-DTSQ$^{\bullet-}$)
II	III	HS	0	FM	HS-Co^{III}(3,5-DTCat^{2-})(3,5-DTSQ$^{\bullet-}$)$_{FM}$
III	III	HS	0	AFM	HS-Co^{III}(3,5-DTCat^{2-})(3,5-DTSQ$^{\bullet-}$)$_{AFM}$

IV (metastable)	II	LS	FM	FM	LS-Co^{II}(3,5-DTSQ$^{\bullet-}$)$_{FM}$(3,5-DTSQ$^{\bullet-}$)$_{FM}$
V	II	LS	AFM	FM	LS-Co^{II}(3,5-DTSQ$^{\bullet-}$)$_{FM}$(3,5-DTSQ$^{\bullet-}$)$_{AFM}$
VI	II	LS	AFM	AFM	LS-Co^{II}(3,5-DTSQ$^{\bullet-}$)$_{AFM}$(3,5-DTSQ$^{\bullet-}$)$_{AFM}$
VII (hs)	II	HS	FM	FM	HS-Co^{II}(3,5-DTSQ$^{\bullet-}$)$_{FM}$(3,5-DTSQ$^{\bullet-}$)$_{FM}$
VIII	II	HS	AFM	FM	HS-Co^{II}(3,5-DTSQ$^{\bullet-}$)$_{AFM}$(3,5-DTSQ$^{\bullet-}$)$_{FM}$
IX	II	HS	AFM	AFM	HS-Co^{II}(3,5-DTSQ$^{\bullet-}$)$_{AFM}$(3,5-DTSQ$^{\bullet-}$)$_{AFM}$

Table S11. B3LYP KS-DFT spin densities on the cobalt center and ligands and energy differences relative to the most stable configuration

Guess	low-temperature structure				high-temperature structure			
	Mulliken spin			$E - E_{\mathrm{I}}$	Mulliken spin			$E - E_{\mathrm{VII}}$
	Co	L1	L2	ΔE (eV)	Co	L1	L2	ΔE (eV)
I	0.04	0.48	0.48	0.0000	0.01	0.50	0.50	0.1377
II	2.86	1.01	1.01	1.7208	2.81	1.05	1.05	0.0069
III	1.81	0.64	0.64	1.4160	2.76	1.00	−0.84	0.1084
IV	1.81	0.64	0.64	1.4160	1.10	0.92	0.92	0.2643
V	0.04	0.48	0.48	0.0000	1.87	−0.38	−0.38	0.9816
VI	0.04	0.48	0.48	0.0000	−0.94	1.02	1.02	1.2407
VII	2.86	1.00	1.00	1.7055	2.81	1.05	1.05	0.0000
VIII	Unconv.				2.76	−0.84	1.00	0.1084

IX	1.73	−0.33	−0.33	1.5266	2.65	−0.86	−0.86	0.2609

However, the computational complexity of the exact–exchange term in a system with periodic boundary conditions (PBCs) limits the applicability of a hybrid GGA functional (e.g., B3LYP, PBE0) to such large systems. The GGA+U method adds an artificially effective on-site Coulomb repulsion term (*U*) to describe systems with localized *d*- and *f*-electrons(20) and provides a possible solution to this problem. This method successfully predicted the insulator behavior of the NiO system rather than the metallic behavior predicted by pure GGA. Ueff plays a role in opening the bandgap, turning partially filled bands into two sets of fully filled bands that are energy-separated.

Determining a suitable *U* for an extended CoL_2 system requires a fair description of the low- and high-spin states, as described above. CoL_2 units with low- and high-temperature molecular structures were placed in a 20 × 24 × 20 Å cell capable of separating two adjacent fragments without overlap. A PBE functional with a *U* value of 0–20 eV was applied to the *d* electrons of cobalt. As a requirement, the low-spin state should have the lowest energy in the low-temperature molecular structure, with an analogous condition required for the high-spin state; these conditions are satisfied when 6 eV $\leq U \leq$ 10 eV (Figure S27). To avoid other possible metastable states with energies lower than those of the low-spin state, we selected the lowest possible *U* value (6 eV).

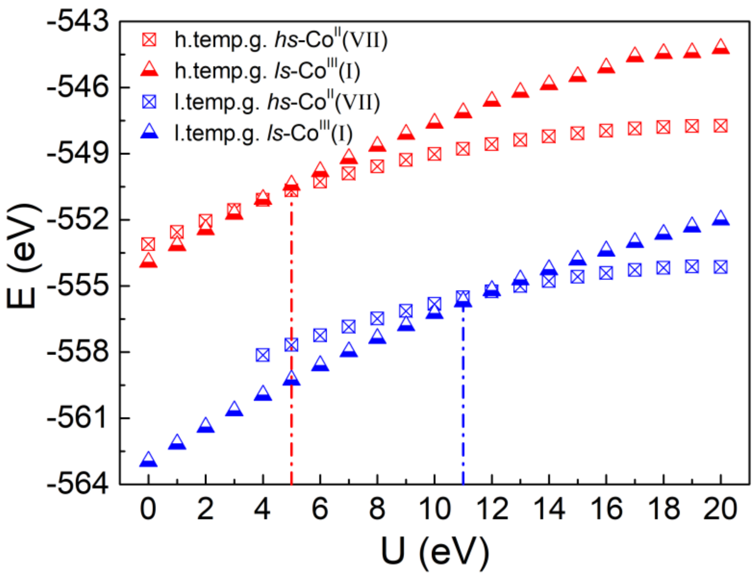

Figure S29. Energies of low-spin- and high-spin-configured CoL_2 in its low- and high-temperature molecular structures as functions of the *U* value applied to the cobalt *d* electrons.

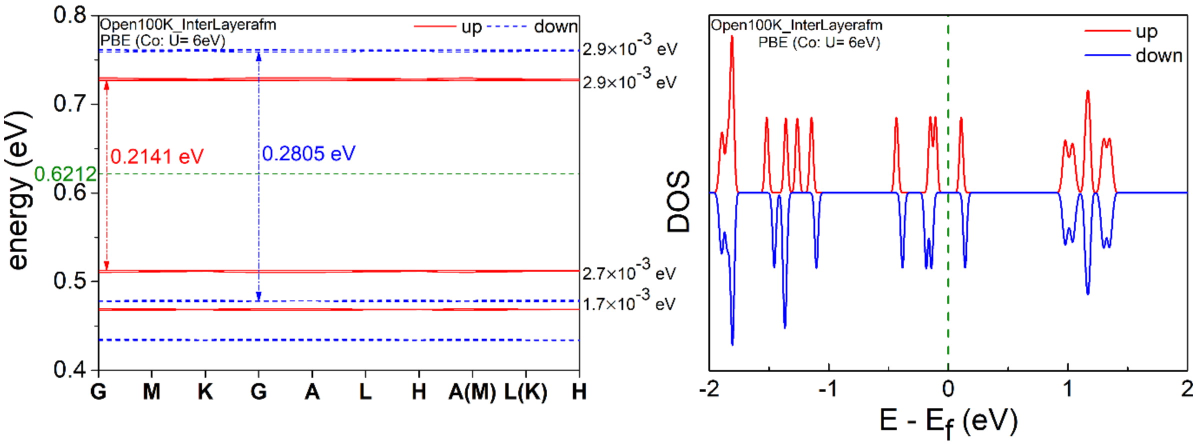

Figure S30. PBE+U band structure (left) and DOS (right) of the low-spin state of **o-1**·$3CH_3CN·H_2O$ in the low-temperature (100 K) crystal structure, with U = 6 eV. The red lines and blue dashed lines in the left panel represent spin-up (α) and spin-down (β), respectively. The green dashed line represents the Fermi level. G(0.0,0.0,0.0); A(0.0,0.0,0.5); L(0.5,0.0,0.5); M(0.5,0.0,0.0); K(1/3,1/3,0.0); H(1/3,1/3,0.5).

To obtain the electronic structures of **o-1**·$3CH_3CN·H_2O$, the band structure and the corresponding DOS were calculated using PBE KS-DFT by applying an effective on-site

Coulomb repulsion of U = 6 eV to the cobalt d electrons. Although small magnetic couplings were observed in this case, the initial magnetic moment was configured using AFM interlayer magnetic ordering for **o-1**·$3CH_3CN{\cdot}H_2O$.

According to the band structure of **o-1**·$3CH_3CN{\cdot}H_2O$, the α component has a flat band structure (0.51 eV) with bands that lie just below the Fermi level and consist of linear combinations of the HOMOs$^{\alpha}$ of CoL_2 in the asymmetric layer (Co_1). The α component bandwidth is 2.7×10^{-3} eV, while the bandwidth of the corresponding β flat band is 1.7×10^{-3} eV. The lower α-component flat band corresponds to the HOMOs$^{\alpha}$ of CoL_2 in the symmetric layer (Co_2). The six bottommost conduction bands with α spins are composed of the LUMOs of open-form DAEpy, with a corresponding bandwidth of 2.9×10^{-3} eV. The six bottommost conduction bands with β spins are composed of the LUMOs$^{\beta}$ of the CoL_2 units, with a corresponding bandwidth of 2.9×10^{-3} eV.

The band structure reveals that **o-1**·$3CH_3CN{\cdot}H_2O$ has semiconductor characteristics, which is inconsistent with the experimental results, as it should be highly conductive. One possible explanation is that the bulky *t*-Bu groups impede effective overlap between the frontier orbitals of the two CoL_2 units, leading to very small bandwidths (10^{-3} – 0.05 eV), and the effective on-site Coulomb repulsion is comparably large. Values of 3.146–3.380 eV for the vacuum on-site Coulomb repulsion *U(v)* were determined for the CoL_2 units, whereas 6.728 eV was determined for open-form 6F-DAE-py_2 based on B3LYP. Although shielding reduces the final effective repulsion, the values should be at least an order of magnitude larger than the bandwidths. Consequently, the electrons should be localized on the fragments; hence, the system moves into a hopping-conductive region that is not captured by the PBE + U-band structure.

Table S12. Plausible guesses for the electronic configurations of singly positively or singly negatively charged CoL_2 units

$[CoL_2]^+$		$[CoL_2]^-$	
I^+	LS-Co^{III}(3,5-DTSQ$^{\bullet-}$)(3,5-DTSQ$^{\bullet-}$)$_{FM}$	I^-	LS-Co^{III}(3,5-DTCat^{2-})(3,5-DTCat^{2-})
II^+	LS-Co^{III}(3,5-DTSQ$^{\bullet-}$)(3,5-DTSQ$^{\bullet-}$)$_{AFM}$	II^-	LS-Co^{III}(3,5-DTCat^{2-})(3,5-DTCat^{2-})
III^+	HS-Co^{III}(3,5-DTSQ$^{\bullet-}$)$_{FM}$(3,5-DTSQ$^{\bullet-}$)$_{FM}$	III^-	LS-Co^{II}(3,5-DTCat^{2-})(3,5-DTSQ$^{\bullet-}$)$_{FM}$
IV^+	HS-Co^{III}(3,5-DTSQ$^{\bullet-}$)$_{FM}$(3,5-DTSQ$^{\bullet-}$)$_{AFM}$	IV^-	LS-Co^{II}(3,5-DTCat^{2-})(3,5-DTSQ$^{\bullet-}$)$_{AFM}$
V^+	HS-Co^{III}(3,5-DTSQ$^{\bullet-}$)$_{AFM}$(3,5-DTSQ$^{\bullet-}$)$_{AFM}$	V^-	HS-Co^{II}(3,5-DTCat^{2-})(3,5-DTSQ$^{\bullet-}$)$_{FM}$
VI^+	HS-Co^{II}(L^0)(3,5-DTSQ$^{\bullet-}$)$_{FM}$	VI^-	HS-Co^{II}(3,5-DTCat^{2-})(3,5-DTSQ$^{\bullet-}$)$_{AFM}$
VII^+	HS-Co^{II}(L^0)(3,5-DTSQ$^{\bullet-}$)$_{AFM}$		

Table S13. Energy differences between singly positively charged CoL_2 in its low spin (100 K) closed molecular structure relative to the most stable configuration ($E - E_{GS}$ (eV)) calculated at various levels of theory and starting from various possible initial guesses.

Guess	PBE	BP86	TPSS	M06-L	TPSSh	B3LYP	HSE06	PBE0	M06-2X
I^+	0.0962	0.0942	0.0675	0.0463	0.0276	0.0158	0.0132	0.0122	0.0053
II^+	0.0000	0.0000	0.0000	0.0000	0.0000	0.0000	0.0000	0.0000	0.0000
III^+	2.7249	2.8288	2.7686	2.0998	2.4334	2.2199	1.8773	1.8235	−3.8727
IV^+	1.6046	1.6424	1.6152	1.4785	1.4725	2.2345	2.0218	1.9683	−3.7817
V^+	0.0962	0.0942	0.0675	0.0463	0.0276	2.1484	2.1775	2.1217	−3.6719
VI^+	1.6046	1.6424	1.6151	1.5264	1.4725	1.3688	1.8965	1.8458	−3.8732
VII^+	0.0962	0.0942	0.0675	0.0463	0.0276	1.4087	1.2401	1.2165	−3.9087

Table S14. Energy differences between singly positively charged CoL_2 in its high spin (380 K) closed molecular structure relative to the most stable configuration ($E - E_{GS}$ (eV)) calculated at various levels of theory and starting from various possible initial guesses.

Guess	PBE	BP86	TPSS	M06-L	TPSSh	B3LYP	HSE06	PBE0	M06-2X
I^+	0.0441	0.0430	0.0280	0.0182	0.0093	0.0052	0.0026	0.0020	Unconv.
II^+	0.0000	0.0000	0.0000	0.0000	0.0000	0.0000	0.0000	0.0000	0.0000
III^+	1.4881	1.5912	1.5332	0.9732	1.1817	0.9795	0.6025	0.5417	−5.0653
IV^+	0.9132	0.9608	Unconv.	0.8517	Unconv.	0.7986	0.4753	0.4241	−3.3880
V^+	0.0441	0.0430	0.0280	0.0182	0.0093	0.6533	0.3512	0.3124	−4.9086
VI^+	0.9132	Unconv.	Unconv.	Unconv.	0.7625	0.7986	0.5488	0.4241	Unconv.
VII^+	0.0441	0.0430	0.0280	0.6798	0.8061	0.6433	0.3394	0.2992	Unconv.

Table S15. Energy differences between singly negatively charged CoL_2 in its low spin (100 K) closed molecular structure relative to the most stable configuration ($E - E_{GS}$ (eV)) calculated at various levels of theory and starting from various possible initial guesses.

Guess	PBE	BP86	TPSS	M06-L	TPSSh	B3LYP	HSE06	PBE0	M06-2X
I^-	0.0000	0.0000	0.0000	0.0000	0.0000	0.0000	0.0000	0.0000	0.0000
II^-	2.1168	2.2061	2.1451	1.6170	1.8207	1.6745	1.3653	1.3251	−4.3751
III^-	Unconv.	1.1273	1.1590	1.1484	1.1171	Unconv.	0.9853	Unconv.	0.8518
IV^-	0.0000	0.0000	0.0000	0.0000	0.0000	0.0000	0.0000	0.0000	1.0577
V^-	2.1168	2.2061	2.1393	1.6170	1.8207	1.6745	1.3653	1.3251	−4.3751
VI^-	1.1041	1.1273	1.1590	1.1484	1.1171	1.1008	0.9853	1.2335	Unconv.

Table S16. Energy differences between singly negatively charged CoL_2 in its high spin (380 K) closed molecular structure relative to the most stable configuration ($E - E_{GS}$ (eV)) calculated at various levels of theory and starting from various possible initial guesses.

Guess	PBE	BP86	TPSS	M06-L	TPSSh	B3LYP	HSE06	PBE0	M06-2X
I^-	0.0000	0.0000	0.0000	0.0000	0.0000	0.0000	0.0000	0.0000	0.0000
II^-	0.5414	0.6299	0.5403	0.0464	0.1990	0.0624	−0.2512	−0.2776	−2.6481
III^-	0.2877	0.3118	0.3056	0.2352	0.4923	Unconv.	0.0638	Unconv.	−1.8075
IV^-	0.0000	0.0000	0.0000	0.0000	0.0000	Unconv.	Unconv.	0.0000	Unconv
V^-	0.5414	0.6299	0.5403	0.0464	0.1990	0.0624	Unconv.	−0.2776	−4.4365
VI^-	0.2877	0.3118	0.3056	0.2352	0.4807	Unconv.	0.0484	0.0399	−2.4288

Because the band structure failed to explain the conductivity mechanism, transport is expected to be dominated by a hopping mechanism. In this case, a charge is momentarily localized on a site, which enables the local system (i.e., CoL_2, 6F-DAE-py_2) to relax to a new lower-energy electronic configuration. The charged configurations of the bridging 6F-DAE-py_2 ligand are trivial: an electron is removed from the HOMO or added to the LUMO. However, more possible configurations exist for the CoL_2 unit because of the presence of multiple redox centers with various magnetic couplings, as listed in Table S12.

The values of the vacuum on-site Coulomb repulsion $U(v)$ of a molecule can be estimated from the ionization potential (IP) and electron affinity (EA) using the following equation:(23)

$$U(v) = \mathrm{IP} - \mathrm{EA}\,;\ \mathrm{IP} = E(n-1) - E(n)\,;\ \mathrm{EA} = E(n) - E(n+1)$$

The $U(v)$ values of the CoL_2 units can also be calculated, as long as the most stable configuration of each charged state is known, as described above. $U(v)$ values were determined to range from 3.146 to 3.380 eV using B3LYP KS-DFT (Table S17).

Table S17. B3LYP KS-DFT calculated vacuum on-site Coulomb repulsions (*U(v)*) determined from IP and EA values of low-temperature (100 K) molecular structures

Molecule	*U(v)* (eV)
CoL_2 (**o-1**·$3CH_3CN·H_2O$, Co_1)	3.380
CoL_2 (**o-1**·$3CH_3CN·H_2O$, Co2)	3.146
6F-DAE-py_2 (**o-1**·$3CH_3CN·H_2O$)	6.728

To reveal the mechanism responsible for electronic conductivity and the peak conductivity observed for **o-1**·$3CH_3CN·H_2O$ (Figure 5b), the electron-transfer rate defined by Marcus theory provides an alternative explanation:(24) it depends on the electron-transfer matrix element (ET) between the reactant state ($A^{+/-}$ + B, a charge localized on A) and the product state (A + $B^{+/-}$, a charge localized on B), the nuclear reorganization energy (λ), and the temperature (T). The electron-transfer matrix element (also referred to as the "electronic coupling energy") can be calculated using a module implemented in the Northwest Computational Chemistry Package (NWChem 6.8),(25) with 6-31G** and 6-311G** basis sets used for cobalt atoms and other atoms, respectively. The rCAM-B3LYP range-separated hybrid functional was used to stabilize the charge on the site.

High-spin-state CoL_2 units are mainly present at high temperatures, while low-spin-state units dominate at low temperatures. At a certain temperature, neutral CoL_2 units with their corresponding spin states were the dominant species in the background crystal-lattice environment. Owing to the nature of a semiconductor, only a very small number of CoL_2 units are thermally excited to charged states, which are embedded in the background. Here, only singly charged cases are considered. Singly positively and negatively charged CoL_2 units with

various electronic configurations must be considered, as the charge carrier can either be a hole or an electron. Possible configurations of charge-transfer pairs are listed in Table S18.

Table S18. Possible electronic configurations of a CoL_2 pair during charge transfer

Configuration Case	Neutral unit	Charged unit	Intermolecular coupling	Charge
1	HS-Co^{II}(3,5-DTSQ$^{\cdot-}$)$_{FM}$(3,5-DTSQ$^{\cdot-}$)$_{FM}$ (high temperature)	LS-Co^{III}(3,5-DTSQ$^{\cdot-}$)(3,5-DTSQ$^{\cdot-}$)$_{AFM}$	AFM	1
2		LS-Co^{III}(3,5-DTSQ$^{\cdot-}$)(3,5-DTSQ$^{\cdot-}$)$_{AFM}$	FM	1
3		LS-Co^{III}(3,5-DTSQ$^{\cdot-}$)(3,5-DTSQ$^{\cdot-}$)$_{FM}$	AFM	1
4		LS-Co^{III}(3,5-DTSQ$^{\cdot-}$)(3,5-DTSQ$^{\cdot-}$)$_{FM}$	FM	1
5		HS-Co^{II}(3,5-DTCat^{2-})(3,5-DTSQ$^{\cdot-}$)$_{FM}$	AFM	-1
6		HS-Co^{II}(3,5-DTCat^{2-})(3,5-DTSQ$^{\cdot-}$)$_{FM}$	FM	-1
7		LS-Co^{III}(3,5-DTCat^{2-})(3,5-DTCat^{2-})		-1
8	LS-Co^{III}(3,5-DTCat^{2-})(3,5-DTSQ$^{\cdot-}$) (low temperature)	LS-Co^{III}(3,5-DTSQ$^{\cdot-}$)(3,5-DTSQ$^{\cdot-}$)$_{AFM}$	AFM	1
9		LS-Co^{III}(3,5-DTSQ$^{\cdot-}$)(3,5-DTSQ$^{\cdot-}$)$_{AFM}$	FM	1
10		LS-Co^{III}(3,5-DTSQ$^{\cdot-}$)(3,5-DTSQ$^{\cdot-}$)$_{FM}$	AFM	1
11		LS-Co^{III}(3,5-DTSQ$^{\cdot-}$)(3,5-DTSQ$^{\cdot-}$)$_{FM}$	FM	1
12		LS-Co^{III}(3,5-DTCat^{2-})(3,5-DTCat^{2-})		-1

Electron-transfer matrix elements between two adjacent or 6F-DAE-py$_2$-connected CoL_2 units with the above configurations were calculated, as shown in Table S19. Only a few observations are made here. Configuration ***6*** (Table S15) is the only possible arrangement when the neutral unit is in a high-spin state and the carrier is an electron, which also indicates that the matrix element is zero when the spin states of the two cobalt centers are different.

The matrix elements of the units in the symmetric layer (Co_2–Co_2) are larger than those in the asymmetric layer (Co_1–Co_1-t1/t2/t3) or in two different layers (Co_1–Co_2-t1/t2/t3) of the low-spin neutral unit in **o-1**·3CH_3CN·H_2O. Co1–Co1-t3 pairs of CoL_2 for the high-spin neutral unit also show comparably high values, but they can only form local triangles; hence, electron transfer between Co1–Co1-t2 pairs limits the rate (Figure S29), which indicates that charge-hopping events mainly occur in the symmetric CoL_2 layer (Co_2) and that **o-1**·3CH_3CN·H_2O behaves as a 2D semiconductor.

Table S19. Electron-transfer elements for various electronic configurations of adjacent pairs

**High temperature configuration ET elements (eV)**							
**CoL_2 pair**	***1***(hole)	***2***(hole)	***3***(hole)	***4***(hole)	***5***(elec)	***6***(elec)	***7***(elec)
**Co_1–Co_1-t1**	0.0000	0.0000	0.0000	0.0000	0.0000	0.0001	0.0000
**Co_1–Co_1-t2**	0.0000	0.0000	0.0000	0.0000	0.0000	0.0725	0.0000
**Co_1–Co_1-t3**	0.0000	0.0000	0.0000	0.0000	0.0000	0.2044	0.0000
**Co_1–Co_2-t1**	0.0000	0.0000	0.0000	0.0000	0.0000	0.0001	0.0000
**Co_1–Co_2-t2**	0.0000	0.0000	0.0000	0.0000	0.0000	0.0001	0.0000
**Co_1–Co_2-t3**	0.0000	0.0000	0.0000	0.0000	0.0000	0.0105	0.0000
**Co_2–Co_2**	0.0000	0.0000	0.0000	0.0000	0.0000	0.1515	0.0000
**Low temperature configuration ET elements (eV)**							
**CoL_2 pair**	***8***(hole)	***9***(hole)	***10***(hole)	***11***(hole)	***12***(elec)		
**Co_1–Co_1-t1**	0.0004	0.0004	0.0000	0.1642	0.0125		
**Co_1–Co_1-t2**	0.0001	0.0000	0.0000	0.0000	0.0321		
**Co_1–Co_1-t3**	0.0006	0.0000	0.0000	0.0001	0.0069		
**Co_1–Co_2-t1**	0.1322	0.0000	0.0000	0.0000	0.0146		
**Co_1–Co_2-t2**	0.0109	0.0641	0.0000	0.0000	0.6780		
**Co_1–Co_2-t3**	0.0018	0.0000	0.0000	0.0068	0.0567		
**Co_2–Co_2**	1.5395	0.1161	0.0000	0.2702	0.9400		

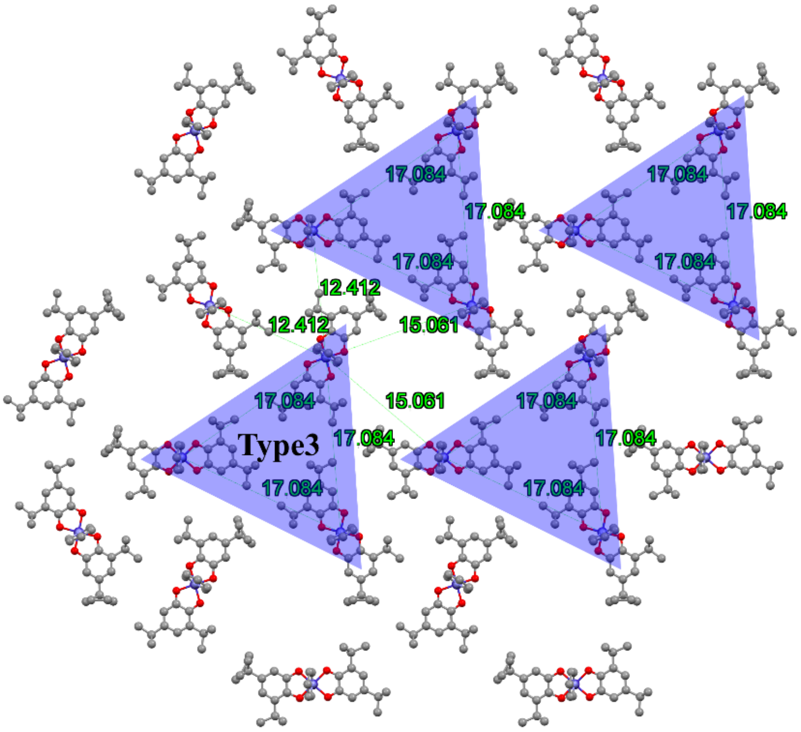

Figure S31. The asymmetric CoL_2 layer in **o-1**·$3CH_3CN·H_2O$ at 100 K shows type-3 pairs (Co_1–Co_1-t3) that can only form local conductive triangles. Color code: Co, dark violet; O, red; N, blue; C, gray; H, white.

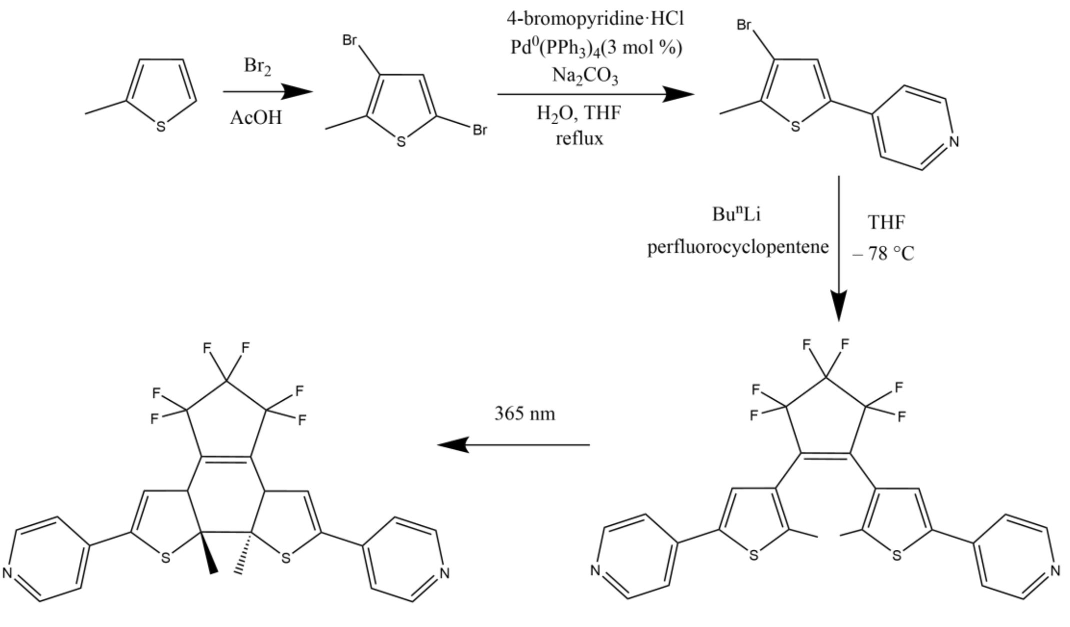

Scheme S1. Synthesis of the open- and closed-form ligands was performed according to previously reported procedures.

References

1. Witt, A, Heinemann, FW, Sproules, S, *et al.* Modulation of magnetic properties at room temperature: coordination-induced valence tautomerism in a cobalt dioxolene complex. *Chem Eur J* 2014; **20**: 11149-11162.
2. Sato, D, Shiota, Y, Juhász, G, *et al.* Theoretical study of the mechanism of valence tautomerism in cobalt complexes. *J Phys Chem A* 2010; **114**: 12928-12935.
3. Swart, M, Groenhof, AR, Ehlers, AW, *et al.* Validation of exchange-correlation functionals for spin states of iron complexes. *J Phys Chem A* 2004; **108**: 5479-5483.
4. Swart, M, Ehlers, AW, Lammertsma, *et al.* Performance of the OPBE exchange-correlation functional. *Mol Phys* 2004; **102**: 2467-2474.
5. Becke, AD. Density-functional thermochemistry. III. The role of exact exchange. *J Chem Phys* 1993; **98**: 5648-5652.
6. Lee, C, Yang, W, Parr, RG. Development of the Colle-Salvetti correlation-energy formula into a functional of the electron density. *Phys Rev B* 1988; **37**: 785-789.
7. Salomon, O, Reiher, M, Hess, BA. Assertion and validation of the performance of the B3LYP* Functional for the first transition mmetal row and the G2 test set. *J Chem Phys* 2002; **117**: 4729-4737.
8. Reiher, M, Salomon, O, Artur Hess, B. Reparameterization of hybrid functionals based on energy differences of states of different mltiplicity. *Theoret Chim Acta* 2001; **107**: 48-55.
9. Borgogno, A, Rastrelli, F, Bagno, A. Predicting the spin state of paramagnetic iron complexes by DFT calculation of proton NMR spectra. *Dalton Trans* 2014; **43**: 9486-9496.
10. Pavlova, NA, Poddel'sky, AI, Bogomyakov, AS, *et al.* New high-spin bis-o-semiquinonato cobalt(II) complexes with neutral donor ligands. *Inorg Chem Commun* 2011; **14**: 1661-1664.
11. Protasenko, NA, Poddel’sky, AI, Bogomyakov, AS, *et al.* Bis-o-semiquinonato complexes of transition metals with 5,7-di-tert-butyl-2-(pyridine-2-yl)benzoxazole. *Polyhedron* 2013; **49**: 239-243.
12. Comba, P. *Modeling of molecular properties*. Weinheim, Germany: Wiley-VCH Verlag GmbH & Co. KGaA.; 2011.
13. Frisch, MJ, Trucks, GW, Schlegel, HB, *et al.* Gaussian 16 Rev. C.01. Wallingford, CT; 2016.
14. Perdew, JP, Burke, K, Ernzerhof, M. Generalized gradient approximation made simple. *Phys Rev Lett* 1996; **77**: 3865-3868.
15. Kresse, G, Hafner, J. Ab initio molecular dynamics for liquid metals. *Phys Rev B* 1993; **47**: 558-561.
16. Kresse, G, Hafner, J. Ab initio molecular-dynamics simulation of the liquid-metal-amorphous-semiconductor transition in germanium. *Phys Rev B* 1994; **49**: 14251-14269.
17. Kresse, G, Furthmüller, J. Efficiency of ab-initio total energy calculations for metals and semiconductors using a plane-wave basis set. *Comput Mat Sci* 1996; **6**: 15-50.

18. Kresse, G, Furthmuller, J. Efficient iterative schemes for ab initio total-energy calculations using a plane-wave basis set. *Phys Rev B* 1996; **54**: 11169-11186.
19. Blochl, PE. Projector augmented-wave method. *Phys Rev B* 1994; **50**: 17953-17979.
20. Floris, A, de Gironcoli, S, Gross, EKU, *et al.* Vibrational properties of MnO and NiO from DFT+U-based density functional perturbation theory. *Phys Rev B* 2011; **84**: 161102.
21. Lu, T, Chen, F. Multiwfn: a multifunctional wavefunction analyzer. *J Comput Chem* 2012; **33**: 580-592.
22. Humphrey, W, Dalke, A, Schulten, K. VMD: visual molecular dynamics. *J Mol Graph* 1996; **14**: 33-38.
23. Demiralp, E, Goddard, WA. Conduction properties of the organic superconductorκ−$(BEDT-TTF)_2Cu(NCS)_2$based on hubbard-unrestricted-Hartree-Fock band calculations. *Phys Rev B* 1997; **56**: 11907-11919.
24. Marcus, RA. On the theory of oxidation-reduction reactions involving electron transfer. I. *J Chem Phys* 1956; **24**: 966-978.
25. Apra, E, Bylaska, EJ, de Jong, WA, *et al.* NWChem: past, present, and future. *J Chem Phys* 2020; **152**: 184102.
